# AnnoLnc: a web server for systematically annotating novel human lncRNAs

**DOI:** 10.1186/s12864-016-3287-9

**Published:** 2016-11-16

**Authors:** Mei Hou, Xing Tang, Feng Tian, Fangyuan Shi, Fenglin Liu, Ge Gao

**Affiliations:** 1 State Key Laboratory of Protein and Plant Gene Research, College of Life Sciences, Center for Bioinformatics, Peking University, Beijing, 100871 P.R. China; 2 Peking-Tsinghua Center for Life Sciences, Academy for Advanced Interdisciplinary Studies, Peking University, Beijing, 100871 P.R. China; 3 Present address: Department of Hematology, St. Jude Children’s Research Hospital, Memphis, TN USA

**Keywords:** Annotation, LncRNAs, Long noncoding RNAs, Transcriptome, Web server

## Abstract

**Background:**

Long noncoding RNAs (lncRNAs) have been shown to play essential roles in almost every important biological process through multiple mechanisms. Although the repertoire of human lncRNAs has rapidly expanded, their biological function and regulation remain largely elusive, calling for a systematic and integrative annotation tool.

**Results:**

Here we present AnnoLnc (http://annolnc.cbi.pku.edu.cn), a one-stop portal for systematically annotating novel human lncRNAs. Based on more than 700 data sources and various tool chains, AnnoLnc enables a systematic annotation covering genomic location, secondary structure, expression patterns, transcriptional regulation, miRNA interaction, protein interaction, genetic association and evolution. An intuitive web interface is available for interactive analysis through both desktops and mobile devices, and programmers can further integrate AnnoLnc into their pipeline through standard JSON-based Web Service APIs.

**Conclusions:**

To the best of our knowledge, AnnoLnc is the only web server to provide on-the-fly and systematic annotation for newly identified human lncRNAs. Compared with similar tools, the annotation generated by AnnoLnc covers a much wider spectrum with intuitive visualization. Case studies demonstrate the power of AnnoLnc in not only rediscovering known functions of human lncRNAs but also inspiring novel hypotheses.

**Electronic supplementary material:**

The online version of this article (doi:10.1186/s12864-016-3287-9) contains supplementary material, which is available to authorized users.

## Background

Long noncoding RNAs (lncRNAs) are operationally defined as RNA transcripts that are 1) longer than 200 nt and 2) do not encode proteins [1]. With high-throughput screening and follow-up experimental validation, several studies show that lncRNAs play essential roles in almost every important biological process, including imprinting [2], cell cycles [3], tumorigenesis [4] and pluripotency maintenance [5] through multiple mechanisms, such as guides, scaffolds, and decoys, as well as chromatin architecture organizers [6, 7].

In recent years, the repertoire of human lncRNAs has rapidly expanded. Approximately 50% of human lncRNAs in the GENCODE catalog were identified in the past five years (15,512 in GENCODE v7 increased to 28,031 in GENCODE v24) [8, 9]. A recent study identified more than 30,000 additional unannotated human lncRNAs genes [10]. However, the functional roles of lncRNAs remain largely elusive: less than 1% of identified human lncRNAs have been experimentally investigated [11], driving the need for computational methods.

Several studies have proposed methods for *in silico* prediction of the function of novel lncRNAs. The “guilt-by-association” strategy is the most widely used approach [12]. A dedicated web server, ncFAN, was developed to predict lncRNA functions based on enriched functional terms of coding genes in the same co-expression module [13, 14]; the algorithm was improved by taking protein-protein interaction into account [15]. Moreover, several attempts have been made to characterize molecules interacting with a given lncRNA [16–20]. The large number and immense functional diversity of lncRNAs call for an integrative annotation tool that incorporates broader spectrum of annotations [6]. Hence, we have developed AnnoLnc, a one-stop annotation portal for novel human lncRNAs with rich annotation and user-friendly interface (see Table 1 for a detailed comparison with similar tools).

**Table 1:** Comparison of AnnoLnc with similar tools

		AnnoLnc	ncFAN	lncPro	lncTar	LongTarget
Annotation	Genomic location	√	-	-	-	-
	RNA secondary structure	√	-	-	-	-
	Expression	RNA-Seq	Microarray	-	-	-
	Regulation	√	-	-	-	-
	Molecular interaction	Protein/RNA	-	Protein	RNA	DNA
	Network	Co-expression	Bi-color	-	-	-
	Function annotation term catalog	GO	GO + KEGG	-	-	-
	Associated traits/diseases	√	-	-	-	-
	Evolution	√	-	-	-	-
Interface	Text summarization	√	-	-	-	-
	Integrative visualization	√	-	-	-	-
	Web-based	√	√	√	-	√
	Mobile-friendly	√	-	-	-	-
	Web Service APIs	√	-	-	-	-

Feature comparison with existing tools suggests AnnoLnc as the most comprehensive annotation tool with rich supports on user interface

## Implementation

**Fig. 1:**
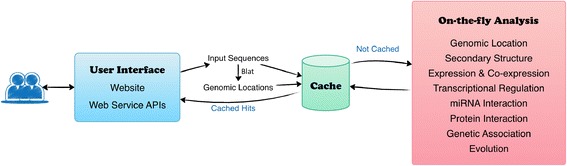
The architecture of the AnnoLnc web server. Users can submit RNA sequences on the AnnoLnc website or by Web Service APIs. First, AnnoLnc tries to map input sequences to the human genome and, if possible, obtain genomic locations. Then, AnnoLnc searches the global cache for possible hits based on both sequences and aligned splicing structures. Cached results are returned directly if a hit is detected, and novel sequences and loci are sent to on-the-fly analysis modules

Designed as a flexible platform, AnnoLnc consists of multiple annotation modules (see Fig. 1 for the architecture of AnnoLnc).

### Genomic location

When a lncRNA is submitted online, AnnoLnc first identifies its genomic coordinate and splicing structure by aligning the input sequence to the human reference genome hg19 with Blat [21]. When a single sequence is aligned in multiple places, genome-wide best alignments are identified by standard pslSort and pslReps. In case of false-positive junction sites caused by mismatches or small indels, putative exons shorter than 20 bp (as well as putative introns shorter than 40 bp) are discarded. The derived coordinates are further compared with annotated human gene models compiled from lncRNAdb [11] and GENCODE [8] (refer to the “Pre-calculated expression profiles of known transcripts” section), and a direct link to the corresponding database entry is provided for hits. Moreover, a link to the UCSC genome browser [22] shows the genomic context of each lncRNA.

### Secondary structure

The structure of an RNA molecule is essential for its biological functions. For each input lncRNA, RNAfold v2.0.7 in the ViennaRNA package [23] is employed to predict the secondary structures, with the option “--noLP” enabled to avoid undesirable isolated base pairs. When multiple candidates are available, the one with minimum free energy is kept (as recommended by the authors of the ViennaRNA package) and rendered online as an interactive plot.

Biological functions of secondary structures lead to local stability and bring evolutionary constraints onto the sequences of lncRNAs [24]. To help users identify functional motifs, AnnoLnc allows users to color each base in the structure plot by its corresponding entropy or conservation score (Fig. 3a and b).

### Expression profile and co-expression-based functional annotation

A transcript’s expression pattern also provides important hints about its functionality [12]. For each input lncRNA, the expression profile is online estimated based on 64 RNA-Seq datasets covering 34 normal samples (16 adult healthy tissues and one embryonic stem cell line with two replicates of each) and 30 cancer cell lines (10 common cancers), then presented in interactive charts (Fig. 3d). Specifically, we mapped the reads of 34 normal samples to human genome hg19 by TopHat (v1.4.1.1). Bam files of 30 cancer samples were downloaded directly from CGHub. (see Additional file 1: Table S3 and S4 for the number of mapped reads and CGHub IDs, respectively.) To improve the response time, the expression of known lncRNAs (including lncRNAs in GENCODE v19 and lncRNAdb v2) was pre-calculated and loaded into the global cache. For novel lncRNAs, we adopt the LocExpress method to perform on-the-fly expression estimation accurately and efficiently.

#### Pre-calculated expression profiles of known transcripts

We generated a gene model (GM) gtf file (http://annolnc.cbi.pku.edu.cn/about/annolnc_gene_model_v1.gtf.gz) covering human lncRNAs in lncRNAdb v2.0 [11] based on GENCODE [8] v19. First, we downloaded human lncRNA sequences in the lncRNAdb and obtained transcript structures as described under the “Genomic location” section. These transcript structures were compared with GENCODE v19 by Cuffcompare (v2.1.1). If the code was “=” or “c”, the lncRNA was replaced by the known transcript; otherwise it was considered a “novel transcript” and merged into GENCODE v19. The expression of all annotated transcripts in the GM file was pre-calculated by StringTie (v1.0.4) with the options “-e -b”, and then normalized by the geometric method in normal and cancer samples separately.

#### On-the-fly expression estimation of input transcripts

Taking advantage of the local nature of RNA-Seq, we developed a novel quantification method called LocExpress for real-time estimation of the expression level based on pre-mapped reads. Briefly, LocExpress takes full advantage of the locality of RNA-Seq data, and makes the abundance calls increasingly. For a novel transcript, LocExpress will first infer its minimum spanning bundle (MSB), and make the expression call based on reads within the MSB only. Then, the estimated relative abundance is further adjusted and normalized, and reported in canonical FPKM unit. (Refer to Hou et al*.* [25] for more details). For the normal sample set, the FPKM of two replicates of each tissue/cell line are averaged to report to users.

#### Co-expression analysis

To help users identify co-regulated partners of the input lncRNA, AnnoLnc reports co-expressed genes based on normal samples and cancer samples. An expression-based functional prediction is further performed by identifying statistically significant enriched Gene Ontology (GO) terms based on co-expressed protein-coding genes. Adjusted *p* values for the multiple-testing issue are reported as well (Fig. 3e) [12, 26].

Specifically, 34 normal samples and 30 cancer samples were treated separately. To avoid the duplicated GO annotation for isoforms, we first obtained expression profiles at the gene level by adding the FPKM of all transcripts of each gene in the GM file. Then, we filtered these genes as described below, resulting in 29,798 genes in the normal sample set and 25,449 genes in the cancer sample set.

1. FPKM filter. The sum of FPKM in all samples should be not less than 1.
2. Tissue-specific filter. The tissue-specific score is calculated by the “getsgene” function in the R package rsgcc. If a gene has a score larger than 0.85, it is not considered fit for the co-expression analysis.

For submitted transcripts that pass the above filters, the Pearson correlations with genes are calculated. Then, highly correlated genes are reported by a “gradually decreased” criterion to remove putative false positives and retain true positives. If there are more than 10 genes with r ≥ 0.9, GO enrichment analysis is performed with these genes directly. If not, we determine whether there are 10 genes above the cutoff of 0.8. This process continues until the cutoff arrives at 0.7. Negatively correlated genes are identified in a similar manner. GO enrichment analysis for these correlated genes is further conducted with the R package GOstats, and significantly enriched GO terms (adjusted *p* value ≤ 0.01, users can also change the cutoff instantly at the result page) are reported as putative functional assignments of the input transcript.

### Transcriptional regulation

Transcriptional factors (TF) largely determine the expression level of lncRNAs. AnnoLnc integrated 498 ChIP-Seq datasets covering 159 (TFs) in 45 cell lines (see Additional file 1: Table S5 for more details). Uniform peak files generated by the ENCODE project were downloaded from http://hgdownload.cse.ucsc.edu/goldenPath/hg19/encodeDCC/wgEncodeAwgTfbsUniform/. AnnoLnc locates the binding sites of 159 TFs in the input lncRNA locus, and reports these binding sites based on their relative location to the lncRNA locus, such as “upstream transcriptional start site (TSS)” (defined as 5Kb upstream), “overlap with TSS”, “inside the lncRNA loci”, “overlap with transcriptional end site (TES)” (defined as 1Kb downstream) and “downstream TES” (Additional file 1: Table S1a). Moreover, we support the *ClosestGene* method as being suggested by Sikora-Wohlfeld et al*.* [27] which could be enabled by the option “Assign peaks to the closest gene” at the result page.

### miRNA interaction

Interacting with miRNAs, lncRNAs can be post-transcriptionally regulated or act as decoys [28]. AnnoLnc provides predicted miRNA family partners of lncRNAs by TargetScan v6.0 [29]. To reduce the potential false positive rate, we run the prediction on 87 highly conserved miRNA families (Additional file 1: Table S6) derived from miRcode [30] (http://www.mircode.org/download.php). Then, conservation scores in primate, mammal and vertebrate clades for each identified site are calculated as described in Jeggari et al*.* [30]*.* For example, 10 species in primates are used in the TargetScan prediction, and if a binding site is identified in eight species, the conservation score in primates is 8/10=0.8. In mammals and vertebrates, scores are calculated in the same manner except that “mammals” are “non-primate mammals” (26 species) and “vertebrates” are “non-mammal vertebrates” (10 species). To further highlight high-confidence sites, predicted sites are screened based on a pre-compiled 61 AGO CLIP-Seq dataset (Additional file 1: Table S7, see the “Calling RNA-protein interactions based on CLIP-Seq data” section for more details about CLIP-Seq analysis), and hit sites are considered “CLIP supported”.

### Protein interaction

lncRNAs can interact with multiple proteins, as guides and/or scaffolds, to perform their functions [31]. For each lncRNA, AnnoLnc reports proteins partners based on both CLIP-Seq data and *in silico* prediction.

#### Calling RNA-protein interactions based on CLIP-Seq data

CLIP-Seq is one of the most widely used high-throughput methods to detect RNA-protein interactions experimentally [32]. AnnoLnc screens putative protein partners for an input lncRNA in 112 CLIP-Seq datasets covering 51 RNA binding proteins (RBPs) other than AGO. In case of methodology bias introduced by heterogeneous analysis pipelines, all the CLIP-Seq data were reanalyzed locally with a uniform pipeline. Finally, protein partners, cell types, treatments and corresponding *p* values reported by the analysis pipeline are reported to users.

Briefly, the raw data of CLIP-Seq datasets were downloaded from the Sequence Read Archive (SRA) (see Additional file 1: Table S8 for a full list). we first trimmed the adapter by FASTX Clipper, and only reads longer than 15 nt were kept and mapped to human genome hg19 by the algorithm BWA-backtrack (v0.7.10-r789) [33] with the options “-n 1 -i 0” (allow one alignment error). Then, only unique mapped reads were kept. To improve precision, we used stringent criteria for site calling with PIPE-CLIP v1.0.0 [34]; FDR cutoffs for both enriched clusters and reliable mutations were set as 0.05 (cross-linking sites in HITS-CLIP data identified by deletion, insertion and substitution were combined).

To evaluate the performance of our pipeline, we downloaded raw reads of wild-type FET proteins (FUS, EWSR1 and TAF15) from DDBJ (SRA025082) and performed the analysis described above. For comparison with reported results (Supplementary Data 1, [35]), cross-linking sites identified by both methods were mapped to RefSeq IDs. Our pipeline shows fairly high precision (0.95 for FUS, 0.96 for EWSR1, and 0.91 for TAF). Meanwhile, we evaluated on a HITS-CLIP dataset for the DGCR8 protein [36]. We downloaded raw reads of all four samples (D8.1, D8.2, T7.1 and T7.2) from GEO (GSE39086) and analyzed them as described above (D8.1 data was excluded because PIPE-CLIP failed to generate cross-linking sites with a “model failed to converge” error). Comparison with the original results downloaded from http://regulatorygenomics.upf.edu/Data/DGCR8/ also shows good precision (0.89 for D8.2, 0.74 for T7.1, and 0.78 for T7.2).

#### Ab initio prediction of lncRNA-protein interaction

AnnoLnc conducts *in silico* prediction across the entire human proteome for each lncRNA by lncPro [17]. We downloaded 99,459 human protein sequences from Ensembl, filtered 1,917 sequences that could not be processed by lncPro (containing “*”, “X”, “U” or length not within 30–30,000 AA), ultimately obtaining 97,542 protein sequences. For efficiency, we modified the source code of lncPro to pre-calculate all protein features in batch. To improve specificity, we further derived the statistical significance of the interaction scores reported by lncPro based on empirical NULL distribution (Additional file 2: Figure S1) generated by random shuffling. Interactions with a *p* value ≤ 0.01 are considered to be significant. Then, the predicted protein partners, interaction scores and empirical *p* values are reported. To make the results more intuitive, Ensembl IDs are finally converted into HGNC gene symbols. If multiple Ensembl IDs are mapped to one gene symbol, the score with the smallest *p* value are reported.

### Genetic association

Large-scale genetic association studies enable detection of multiple phenotypic traits that lncRNAs may associate with [37]. By integrating the NHGRI GWAS Catalog [38] (downloaded from the UCSC genome browser), AnnoLnc links an input lncRNA to diseases/traits based on strong linkage blocks defined by linkage disequilibrium (LD) values in multiple populations. Specifically, AnnoLnc first scans all SNPs within the transcript region (5 Kb upstream to 1 Kb downstream of each input transcript). Using one of these SNPs as an example, a SNP is linked with a tagSNP if it is within the haplotype region (defined as r^2^ > 0.5, ftp://ftp.ncbi.nlm.nih.gov/hapmap/ld_data/2009-04_rel27/) tagged by the tagSNP reported in the NHGRI GWAS Catalog. Then, this linked SNP, corresponding tagSNPs, traits/diseases, *p* values, significance (defined as *p* value ≤ 5e-8), LD values, populations from which these LD values are derived, as well as supporting PubMed IDs, are reported by AnnoLnc (Additional file 1: Table S2).

### Evolution

The evolutionary signature is an important hint as to biological function. For each submitted lncRNA, we incorporated the 46 way phyloP score (primates, mammals/placentals and vertebrates) from the UCSC Genome Browser, and the derived allele frequency (DAF) [39] of the YRI population (Yoruba in Ibadan, Nigeria) from http://compbio.mit.edu/human-constraint/ for every position (if has corresponding scores) of both the exon and promoter region (1 Kb upstream). To obtain an overall view, AnnoLnc calculates the mean scores for the exon and promoter regions, and organized the scores into interactive bar charts.

Because many lncRNAs are partially conserved, we also report conserved elements predicted by phastCons [40] in different clades with the length and score, which is an indicator of conservation. The phastCons conserved elements were downloaded from ftp://hgdownload.cse.ucsc.edu/goldenPath/hg19/database. The score reported to users is the LOD score. Conserved elements shorter than 20 bp are omitted from the table. These conserved blocks can help users identify functional elements combined with other annotation results, especially in the integrated view (Fig. 3f).

### AnnoLnc website

The AnnoLnc website runs on the Tomcat server. The backend is based on Java Servlet and MySQL database. In the frontend, some JavaScript libraries are used to facilitate accessibility. Bootstrap is used for the mobile friendly layout. jQuery is used for Ajax. DataTables is used to show tables and Highcharts for interactive charts. The display of the interactive SVG plot is enabled by “svg-pan-zoom”, available at https://github.com/ariutta/svg-pan-zoom.

## Results and Discussion

### User Interface

**Fig. 2:**
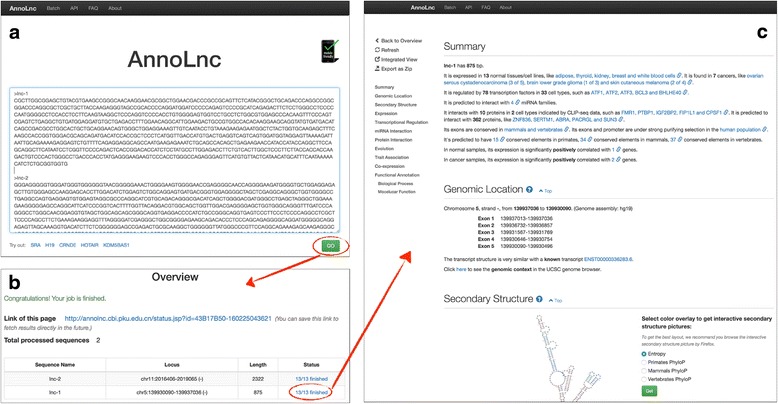
The web interface of AnnoLnc. Only a few clicks are required from submission of sequences to receipt of annotation results. **a** The “Home” page. Users can submit lncRNA sequences here to start the on-the-fly analysis. **b** The “Overview” page of run status and basic information of processed lncRNAs. **c** An example of the annotation result page. Most annotations are presented in this page with a sidebar on the left for quick jump

AnnoLnc is designed to be intuitive. The most common operations (such as submitting sequences and obtaining annotation results) can be performed with just a few clicks. As showed in Figs. 1 and 2, users can submit RNA sequences in fasta format at the “Home” page of AnnoLnc website (Fig. 2a) or by Web Service APIs. AnnoLnc tries to map input sequences to the human genome and, if possible, obtain genomic locations and report them in the “Overview” page (Fig. 2b). Some tune-ups are made to improve the user experience. AnnoLnc first searches the global cache for possible hits based on both sequences and aligned splicing structures. Cached results are returned directly if a hit is detected, and novel sequences and loci are sent to on-the-fly analysis modules (Fig. 1). When users fetch results, annotations generated by each module will be well summarized and integrated into intuitive web pages (Fig. 2c).

**Fig. 3:**
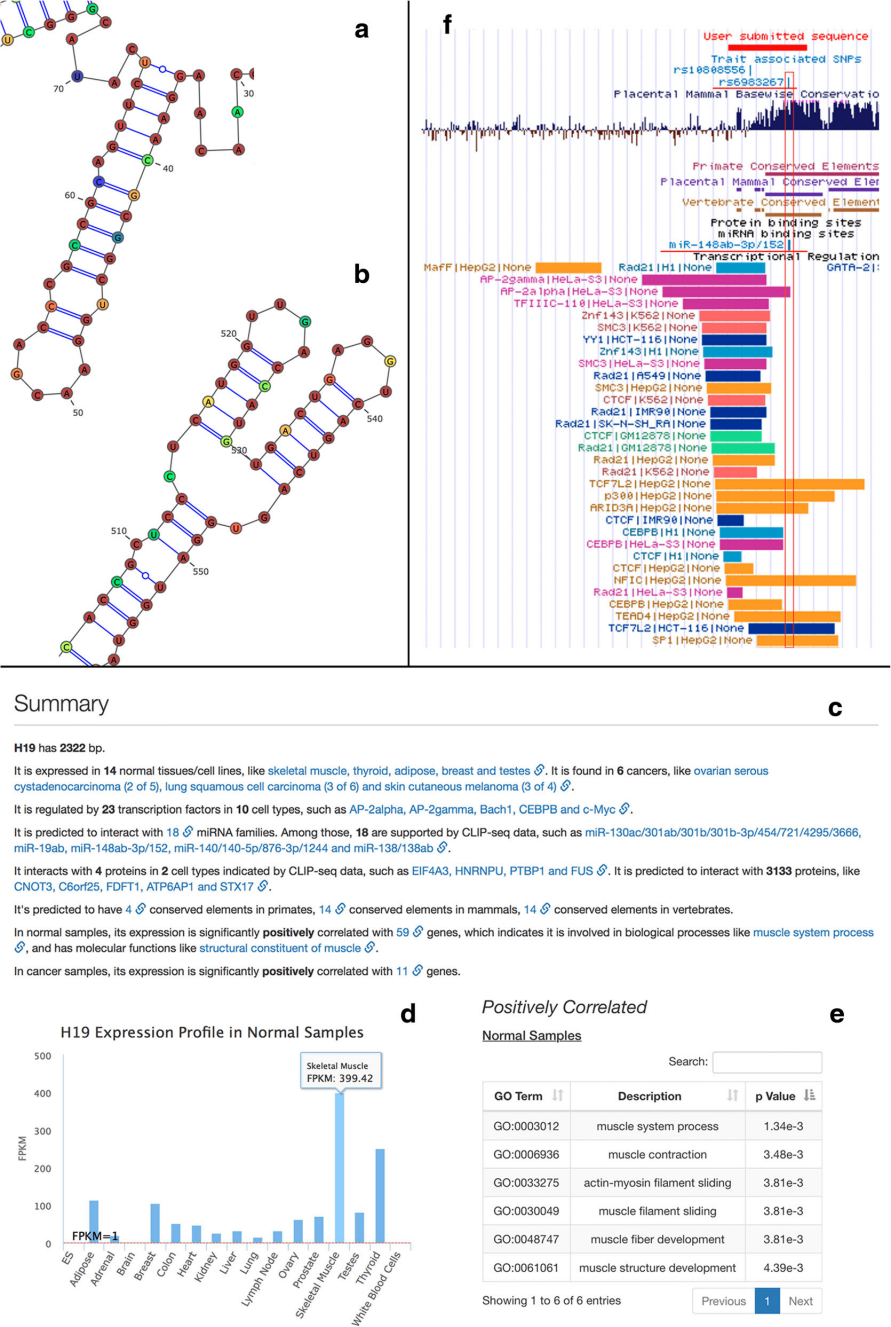
The case studies for AnnoLnc. **a-b** The case study of lncRNA *SRA*. In the interactive secondary structure plot with vertebrate phyloP scores as the color overlay, two sub-structures are very easy to be identified because most bases are colored red. **a** is a hairpin region that corresponds to the most conserved H2 sub-structure highlighted by Novikova et al*.* [42]. **b** is a three-way junction hairpin region that is very similar to the conserved regions H15, H16 and H17 verified by Novikova et al*.* [42]. **c-e** The case study of lncRNA *H19*. **c** is a summary of the annotation results, which helps users quickly grasp the essentials. **d** is the expression profile of *H19* in normal samples. It has the highest expression in “skeletal muscle”. **e** is the predicted GO terms based on positively correlated coding genes in normal samples. The terms are all muscle related. **f** The integrative view of lncRNA *CCAT2* in the UCSC genome browser for annotations at the transcript level. It is easy to determine that rs6983267 is within the seed binding site of the miRNA family miR-148ab-3p/152

The default web interface is implemented based on responsive design, which enables the optimal view for both desktop PCs and mobile devices. To further help users quickly grasp the essentials from abundant annotations generated by various modules, AnnoLnc provides a concise summary text in plain English for each input lncRNA at the top of the annotation result page by abstraction-based summarization, with inline links available for checking original results when necessary (Fig. 3c). Furthermore, AnnoLnc supports exporting transcript-level annotations (including transcript structure, TF binding sites, miRNA binding sites, protein binding sites and SNPs) at the locus of an input lncRNA onto the UCSC genome browser as pre-tuned custom tracks (Fig. 3f).

In addition to the browser-based interactive analysis, AnnoLnc provides a “batch mode” that allows users to upload multiple sequences together and fetch annotations as a ZIP. AnnoLnc also offers a set of JSON-based Web Service APIs (Table 2) to help advanced users run the analysis and fetch results programmatically, enabling an easy integration of AnnoLnc into downstream analysis pipelines (see http://annolnc.cbi.pku.edu.cn/api.jsp for more detailed instruction as well as the demo code).

**Table 2:** The introduction to Web Service APIs provided by AnnoLnc

Name	URL	Parameters	Return	HTTP Method
Upload	http://annolnc.cbi.pku.edu.cn/service/upload	*file*: a file containing lncRNA sequences (less than 500) in fasta format. *token*: a unique ID for authorized submission of batch job through the Web Service*. *email*: your email address used to apply the token.	The job ID.	POST
Info	http://annolnc.cbi.pku.edu.cn/service/info	*id*: the job ID.	The run info of this job, especially the run status for each sequence. Note that you can only get the download URL after all analyses are finished. Please check the run status first before fetching the download URL.	GET, POST
Fetch	http://annolnc.cbi.pku.edu.cn/service/fetch	*submitID*: the job ID. [*seqName*]: the name of a lncRNA which you want to download its annotation results.	The URL of the annotation results in a ZIP package.	GET, POST

* see http://annolnc.cbi.pku.edu.cn/api.jsp#apply-for-token for more details

### Case studies by AnnoLnc

The noncoding form of the steroid receptor RNA activator (*SRA*, AF092038, http://annolnc.cbi.pku.edu.cn/cases/SRA) has been reported to function as a noncoding RNA by Lanz et al*.* [41] and is the first lncRNA that has experimentally derived secondary structure, which was derived by Novikova et al*.* [42]. In the interactive secondary structure plot with vertebrate phyloP score as color overlay, it is easy to identify two conserved regions. One is a hairpin region from base 30 to 72 (Fig. 3a). With approximately 75% of bases colored red, this conserved sub-structure is clearly distinguishable from others. In fact, this region corresponds to the most conserved H2 sub-structure highlighted by Novikova et al*.* [42]. Site-directed mutagenesis of this region reduced the co-activation performance of *SRA* by 40% [43], suggesting the importance of lncRNA secondary structure on its function [44]. The other distinct region is a three-way junction hairpin sub-structure from base 506 to 555 with 78% colored red (Fig. 3b). This region is very similar to the conserved regions H15, H16 and H17 verified by Novikova et al*.* [42].

*H19* (http://annolnc.cbi.pku.edu.cn/cases/H19) is the first identified imprinting lncRNA [45, 46]. Consistent with the work by Dey et al*.* [47], AnnoLnc shows that *H19* has the highest expression in “skeletal muscle” (Fig. 3d) and is associated with muscle-related function terms such as “muscle fiber development” (GO:0048747, Fig. 3e). Moreover, the “transcriptional regulation” module reports that *H19* is regulated by multiple known cell proliferation- and cell cycle-related TFs, including c-Myc, Max, Maz, and E2F6 in cancer cell lines (Additional file 1: Table S1a), confirming its previously reported tumorigenesis function [48]. In addition, AnnoLnc identified 18 CLIP-Seq-supported binding miRNA families (Additional file 1: Table S1b), and several miRNAs have already been verified experimentally, such as miR-138 in colorectal cancer [49] and miR-17-5p in HeLa cells and myoblasts [50].

In addition to confirming previous reports, the integrative annotations provided by AnnoLnc help users to generate new hypotheses. For example, lncRNA *CCAT2* (http://annolnc.cbi.pku.edu.cn/cases/CCAT2) has been reported to promote colorectal cancer (CRC) growth and metastasis [51] (also see Additional file 1: Table S2 for associated diseases), and risk allele G of rs6983267 within the *CCAT2* transcript is associated with up-regulated expression of this lncRNA [51]. Integrating miRNA annotation with the variant track (Fig. 3f), SNP rs6983267 is found to be just within the seed binding site of miRNA family miR-148ab-3p/152, suggesting that SNP rs6983267 might weaken the binding of miR-148ab-3p/152 and increases the transcript level of *CCAT2*.

## Conclusions

To the best of our knowledge, AnnoLnc is the only online web server to systematically annotate novel human lncRNAs. The annotation generated by AnnoLnc covers a much wider range of perspectives with intuitive visualization and summarization. Several case studies have shown the power of AnnoLnc to systematically annotate lncRNAs, as well as inspire novel hypotheses for follow-up experimental studies. Employing Web Service APIs, AnnoLnc is friendly for not only interactive users, but also programmers.

## Availability and requirements

Project name: AnnoLnc

Project home page: http://annolnc.cbi.pku.edu.cn

Operating system: AnnoLnc can be accessed from any platform by using modern Web browsers (recommended but not limited to the latest version of Safari, Chrome and Firefox).

Programming languages: Java, Python, R, Shell and JavaScript.

Any restrictions to use by non-academics: For non-academic use, please contact annolnc@mail.cbi.pku.edu.cn.

## Additional files

Additional file 1: Table S1a. The annotation result of “trancriptional regulation” of IncRNA *H19*. **Table S1b.** The annotation result of “miRNA interaction” of IncRNA *H19*. **Table S2.** The annotation result of “trait association” of IncRNA *CCAT2*. **Table S3.** RNA-Seq datasets of normal samples. **Table S4.** RNA-Seq datasets of cancer samples. **Table S5.** ChIP-Seq datasets. **Table S6.** miRNA families to run targetScan prediction**. Table S7.** CLIP Seq datasets of AGO. **Table S8.** CLIP-Seq datasets of RNA binding protein. (XLS 190 kb)
Additional file 2: Figure S1. The empirical NULL distribution of interaction scores generated by random shuffling (calculated by lncPro). The red line is for the cutoff *p* value (0.01). (DOCX 63 kb)

## Abbreviations

DAF: Derived allele frequency
GM: Gene model
GO: Gene ontology
LD: Linkage disequilibrium
LncRNA: Long noncoding RNA
MSB: Minimum spanning bundle
TES: Transcriptional end site
TF: Transcriptional factor
TSS: Transcriptional start site
