# Supplementary material for: AnnoLnc: a web server for systematically annotating novel human lncRNAs
Source: BMC Genomics. 2016 Nov 16;17:931. doi: 10.1186/s12864-016-3287-9 (PMC5112684; doi:10.1186/s12864-016-3287-9)
Supplement: Additional file 1: Table S1a. — The annotation result of “trancriptional regulation” of IncRNA H19. Table S1b. The annotation result of “miRNA interaction” of IncRNA H19. Table S2. The annotation result of “trait association” of IncRNA CCAT2. Table S3. RNA-Seq datasets of normal samples. Table S4. RNA-Seq datasets of cancer samples. Table S5. ChIP-Seq datasets. Table S6. miRNA families to run targetScan prediction. Table S7. CLIP Seq datasets of AGO. Table S8. CLIP-Seq datasets of RNA binding protein. (XLS 190 kb) [file 12864_2016_3287_MOESM1_ESM.xls]

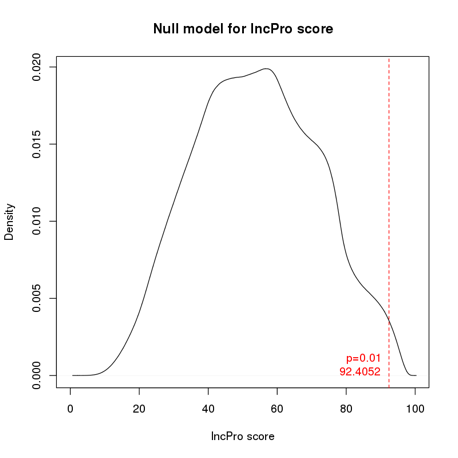


**Figure S1.** The empirical NULL distribution of interaction scores generated by random shuffling (calculated by lncPro). The red line is for the cutoff *p* value (0.01).
