# Supplementary material for: AnnoLnc: a web server for systematically annotating novel human lncRNAs
Source: BMC Genomics. 2016 Nov 16;17:931. doi: 10.1186/s12864-016-3287-9 (PMC5112684; doi:10.1186/s12864-016-3287-9)
Supplement: Supplementary file 2 [file 12864_2016_3287_MOESM2_ESM.docx]

## Sheet: S1a

Table S1 a) The annotation result of "trancriptional regulation" of lncRNA H19
Transcription factor	Cell type	Treatment	Up TSS	Overlap TSS	Inside	Overlap TES	Down TES
AP-2alpha	HeLa-S3	None	Yes	No	No	No	No
AP-2gamma	HeLa-S3	None	Yes	No	No	No	No
Bach1	H1	None	Yes	No	No	No	No
CEBPB	HeLa-S3	None	Yes	No	No	No	No
c-Myc	HeLa-S3	None	Yes	No	No	No	No
	K562	None	Yes	No	No	No	No
	NB4	None	Yes	No	No	No	No
CTCF	GM12878	None	Yes	No	No	No	No
	H1	None	Yes	No	No	No	No
	IMR90	None	Yes	No	No	No	No
	K562	None	Yes	No	No	No	No
E2F6	K562	None	Yes	Yes	Yes	No	No
EBF1	GM12878	None	Yes	No	No	No	No
Egr-1	K562	None	No	No	Yes	No	No
ELK1	GM12878	None	Yes	No	No	No	No
ETS1	K562	None	Yes	No	No	No	No
Max	HeLa-S3	None	Yes	No	No	No	No
	K562	None	Yes	No	Yes	No	No
	NB4	None	Yes	No	No	No	No
MAZ	K562	None	Yes	No	No	No	No
NANOG	H1	None	Yes	No	No	No	No
NRSF	K562	None	No	Yes	No	No	No
	PANC-1	None	No	No	Yes	No	No
Pol2-4H8	PANC-1	None	No	No	No	No	Yes
Rad21	A549	None	Yes	No	No	No	No
	GM12878	None	Yes	No	No	No	No
	H1	None	Yes	No	No	No	No
	HeLa-S3	None	Yes	No	No	No	No
	IMR90	None	Yes	No	No	No	No
	K562	None	Yes	No	No	No	No
	SK-N-SH_RA	None	Yes	No	No	No	No
SETDB1	K562	None	Yes	No	No	No	No
	U2OS	None	Yes	No	No	No	No
SMC3	GM12878	None	Yes	No	No	No	No
	HeLa-S3	None	Yes	No	No	No	No
	K562	None	Yes	No	No	No	No
TAL1	K562	None	Yes	No	No	No	No
USF-1	H1	None	Yes	No	No	No	No
ZBTB7A	K562	None	No	No	No	No	Yes
Znf143	K562	None	Yes	No	No	No	No
	TSS: transcriptional start site
	TES: transcriptional end site

## Sheet: S1b

Table S1 b) The annotation result of "miRNA interaction" of lncRNA H19
miRNA families	start	end	Conservation score			CLIP support
			Primates	Mammals	Vertebrates
miR-130ac/301ab/301b/301b-3p/454/721/4295/3666	2000	2007	0.8	0.58	0	Yes
miR-19ab	1999	2005	0.8	0.54	0	Yes
miR-130ac/301ab/301b/301b-3p/454/721/4295/3666	2014	2020	0.8	0.5	0	Yes
miR-19ab	2013	2019	0.8	0.42	0	Yes
miR-148ab-3p/152	2015	2021	0.7	0.5	0	Yes
miR-140/140-5p/876-3p/1244	1651	1657	0.7	0.42	0	Yes
miR-138/138ab	1818	1824	0.7	0.23	0	Yes
miR-18ab/4735-3p	111	117	0.7	0.15	0	Yes
miR-216b/216b-5p	1948	1954	0.7	0.08	0	Yes
miR-17/17-5p/20ab/20b-5p/93/106ab/427/518a-3p/519d	1642	1649	0.7	0	0	Yes
miR-29abcd	1570	1577	0.6	0.54	0	Yes
miR-1ab/206/613	2216	2222	0.6	0.23	0	Yes
miR-130ac/301ab/301b/301b-3p/454/721/4295/3666	1657	1663	0.6	0	0	Yes
miR-22/22-3p	327	333	0.5	0.12	0	Yes
miR-132/212/212-3p	2296	2302	0.5	0	0	Yes
miR-138/138ab	1347	1353	0.5	0	0	Yes
miR-194	2297	2304	0.5	0	0	Yes
miR-22/22-3p	805	811	0.5	0	0	Yes
miR-24/24ab/24-3p	2072	2078	0.5	0	0	Yes
miR-103a/107/107ab	1637	1644	0.4	0.5	0	Yes
miR-196abc	1660	1666	0.4	0.08	0	Yes
miR-18ab/4735-3p	1456	1462	0.4	0	0	Yes
miR-193/193b/193a-3p	189	195	0.4	0	0	Yes
miR-338/338-3p	81	87	0.3	0.12	0	Yes

## Sheet: S2

Table S2 The annotation result of "trait association" of lncRNA CCAT2
Linked SNP	Tag SNP	Tait	p value	Significant	PubMed	Linkage	Population
rs11985829	rs7014346	Colorectal cancer	0.00000000000000000000000009	Yes	18372901	0.71, 0.95, 0.91, 0.94	CEU, CHB, GIH, MEX
rs10808555	rs7014346	Colorectal cancer	0.00000000000000000000000009	Yes	18372901	0.95, 1	CHD, JPT
rs12682374	rs10505477	Prostate cancer (early onset)	0.000000009	Yes	24740154	1, 0.57	JPT, YRI
rs12682374	rs10505477	Colorectal cancer	0.0000000000008	Yes	24737748	1, 0.57	JPT, YRI
rs12682374	rs10505477	Colorectal cancer	0.00000000003	Yes	17618283	1, 0.57	JPT, YRI
rs12682374	rs6983267	Colorectal cancer	0.00000000000005	Yes	24836286	1	JPT
rs12682374	rs6983267	Prostate cancer	0.000000000000004	Yes	24753544	1	JPT
rs12682374	rs6983267	Prostate cancer (early onset)	0.00000001	Yes	24740154	1	JPT
rs12682374	rs6983267	Colorectal cancer	0.00000000001	Yes	23266556	1	JPT
rs12682374	rs6983267	Prostate cancer	0.000009	No	21743057	1	JPT
rs12682374	rs6983267	Colorectal cancer	0.00000002	Yes	21242260	1	JPT
rs12682374	rs6983267	Colorectal cancer	0.00000000007	Yes	18372905	1	JPT
rs12682374	rs6983267	Prostate cancer	0.0000000000009	Yes	18264097	1	JPT
rs12682374	rs6983267	Prostate cancer	0.000000000007	Yes	18264096	1	JPT
rs12682374	rs6983267	Colorectal cancer	0.00000000000001	Yes	17618284	1	JPT
rs12682374	rs6983267	Prostate cancer	0.0000000000009	Yes	17401363	1	JPT
rs10808556	rs10505477	Prostate cancer (early onset)	0.000000009	Yes	24740154	0.75, 1	ASW, CHB
rs10808556	rs10505477	Colorectal cancer	0.0000000000008	Yes	24737748	0.75, 1	ASW, CHB
rs10808556	rs10505477	Colorectal cancer	0.00000000003	Yes	17618283	0.75, 1	ASW, CHB
rs10808556	rs7014346	Colorectal cancer	0.00000000000000000000000009	Yes	18372901	0.83	TSI
rs6983267	rs10505477	Prostate cancer (early onset)	0.000000009	Yes	24740154	0.92, 0.98, 0.93, 0.95, 0.98	CEU, CHD, GIH, MEX, TSI
rs6983267	rs10505477	Colorectal cancer	0.0000000000008	Yes	24737748	0.92, 0.98, 0.93, 0.95, 0.98	CEU, CHD, GIH, MEX, TSI
rs6983267	rs10505477	Colorectal cancer	0.00000000003	Yes	17618283	0.92, 0.98, 0.93, 0.95, 0.98	CEU, CHD, GIH, MEX, TSI
rs6983267	rs6983267	Colorectal cancer	0.00000000000005	Yes	24836286	1, 1, 1, 1, 1, 1, 1, 1, 1, 1	ASW, CEU, CHB, CHD, GIH, LWK, MEX, MKK, TSI, YRI
rs6983267	rs6983267	Prostate cancer	0.000000000000004	Yes	24753544	1, 1, 1, 1, 1, 1, 1, 1, 1, 1	ASW, CEU, CHB, CHD, GIH, LWK, MEX, MKK, TSI, YRI
rs6983267	rs6983267	Prostate cancer (early onset)	0.00000001	Yes	24740154	1, 1, 1, 1, 1, 1, 1, 1, 1, 1	ASW, CEU, CHB, CHD, GIH, LWK, MEX, MKK, TSI, YRI
rs6983267	rs6983267	Colorectal cancer	0.00000000001	Yes	23266556	1, 1, 1, 1, 1, 1, 1, 1, 1, 1	ASW, CEU, CHB, CHD, GIH, LWK, MEX, MKK, TSI, YRI
rs6983267	rs6983267	Prostate cancer	0.000009	No	21743057	1, 1, 1, 1, 1, 1, 1, 1, 1, 1	ASW, CEU, CHB, CHD, GIH, LWK, MEX, MKK, TSI, YRI
rs6983267	rs6983267	Colorectal cancer	0.00000002	Yes	21242260	1, 1, 1, 1, 1, 1, 1, 1, 1, 1	ASW, CEU, CHB, CHD, GIH, LWK, MEX, MKK, TSI, YRI
rs6983267	rs6983267	Colorectal cancer	0.00000000007	Yes	18372905	1, 1, 1, 1, 1, 1, 1, 1, 1, 1	ASW, CEU, CHB, CHD, GIH, LWK, MEX, MKK, TSI, YRI
rs6983267	rs6983267	Prostate cancer	0.0000000000009	Yes	18264097	1, 1, 1, 1, 1, 1, 1, 1, 1, 1	ASW, CEU, CHB, CHD, GIH, LWK, MEX, MKK, TSI, YRI
rs6983267	rs6983267	Prostate cancer	0.000000000007	Yes	18264096	1, 1, 1, 1, 1, 1, 1, 1, 1, 1	ASW, CEU, CHB, CHD, GIH, LWK, MEX, MKK, TSI, YRI
rs6983267	rs6983267	Colorectal cancer	0.00000000000001	Yes	17618284	1, 1, 1, 1, 1, 1, 1, 1, 1, 1	ASW, CEU, CHB, CHD, GIH, LWK, MEX, MKK, TSI, YRI
rs6983267	rs6983267	Prostate cancer	0.0000000000009	Yes	17401363	1, 1, 1, 1, 1, 1, 1, 1, 1, 1	ASW, CEU, CHB, CHD, GIH, LWK, MEX, MKK, TSI, YRI

## Sheet: S3

Table S3 RNA-Seq datasets of normal samples
Tissues / Cell_lines	ID (GEO / ERA)	# Mapped Reads
ES(H1)	GSE16256_GSM915328	185981389
	GSE16256_GSM915329	193720056
adipose	ERR030880	72016722
	ERR030888	69977356
adrenal	ERR030881	69972253
	ERR030889	68851030
brain	ERR030882	67742190
	ERR030890	56562172
breast	ERR030883	71042232
	ERR030891	70317092
colon	ERR030884	75611101
	ERR030892	73501813
heart	ERR030886	76500047
	ERR030894	69997135
kidney	ERR030885	72454098
	ERR030893	71489732
liver	ERR030887	75691424
	ERR030895	69467433
lung	ERR030879	74782632
	ERR030896	75067237
lymph_node	ERR030878	76254002
	ERR030897	72548644
ovary	ERR030874	76632318
	ERR030901	74261788
prostate	ERR030877	78294550
	ERR030898	78088622
skeletal_muscle	ERR030876	75105888
	ERR030899	77030270
testes	ERR030873	77452265
	ERR030902	76053043
thyroid	ERR030872	77177915
	ERR030903	73248703
white_blood_cells	ERR030875	77200615
	ERR030900	76823560

## Sheet: S4

Table S4 RNA-Seq datasets of cancer samples
Cancer	Cell line	CGHub ID
Brain Lower Grade Glioma	SF539	d39fa32d-07d4-467d-b327-0d11d9fd85ff
	SF268	f399f213-694c-4463-b855-132f433df92d
	SNB75	9b530d80-e6c8-4585-8dc9-99e9a2305e57
Breast Invasive Carcinoma	BT-549	f94eba44-34b1-4fb4-a5d6-584e35c9f473
	MDA-MB-435S	bd30ed4a-1e38-4b26-928c-f0fb20a27181
	MDA-MB-468	0ab4dac4-bbb9-4cd5-ae67-9469c6e8f21b
	MDA-MB-231	7dec127a-54a7-4e3d-bd6d-dab232b3206e
	Hs578T	cef780d4-0a0c-4339-bdbe-02765e8168ba
Chronic Lymphocytic Leukemia	HL-60	5f2b6663-2171-4731-a209-2db8358356c2
Colon Adenocarcinoma	KM12	bde66db2-40f5-4a47-92b4-151050895755
Kidney Renal Clear Cell Carcinoma	A-498	ce9f1b08-8a07-416a-ab43-90e011092b08
	UO-31	3e6ded62-51b1-4168-b651-62bc3ffc9b25
	ACHN	1faa0a53-04e8-4e76-bf36-85623edb1a45
Lung Squamous Cell Carcinoma	NCI-H522	9da8f1f7-d606-4fe8-ab14-c9fe2a4b7afc
	HOP-62	094f1c97-45dd-405c-99ff-1e44ce52cbb9
	HOP-92	6b1b06b6-c931-4106-a3e5-8f8c04c5726e
	NCI-H23	e06d66ca-cd61-4c69-97e2-3da1aaabc57d
	NCI-H322	8fdc43d3-7c5e-4207-b125-409293f7181c
	EKVX	f7bafca2-ca9b-4d6e-a1fa-a1486dc3b30c
Multiple Myeloma	RPMI8226	6b973641-ef81-4981-8798-baf20f6d7924
Ovarian Serous Cystadenocarcinoma	OVCAR-4	f3cc5e3e-54e0-4f63-8ced-3c60bea7aab7
	IGROV1	d08adcda-bcbf-46bd-a700-9af0823d41eb
	OVCAR-8	d16079cf-954f-40fd-bd09-434701481977
	SK-OV-3	fb4feed3-9977-4d74-a987-970f5c760d98
	OVCAR-3	8894a110-521a-44bb-87d6-4c2bb6c1d2a8
Prostate Adenocarcinoma	PC-3	811a96a5-a7f2-4082-9044-e6972c7316a9
Skin Cutaneous Melanoma	Malme-3M	6ad76af6-73c5-4ace-9a4b-649861d2e01d
	UACC-62	095495fa-5647-4dbe-82a3-f639f19689f9
	SK-MEL-28	4a77f393-6c2f-47d7-b7e5-411982bf75a6
	UACC-257	15903736-28e7-4842-9477-584736a7473d

## Sheet: S5

Table S5 ChIP-Seq datasets
Peak file	Cell line	Treatment	Antibody
wgEncodeAwgTfbsBroadDnd41Ezh239875UniPk.narrowPeak.gz	Dnd41	None	EZH2
wgEncodeAwgTfbsBroadGm12878Ezh239875UniPk.narrowPeak.gz	GM12878	None	EZH2
wgEncodeAwgTfbsBroadH1hescChd1a301218aUniPk.narrowPeak.gz	H1	None	CHD1
wgEncodeAwgTfbsBroadH1hescEzh239875UniPk.narrowPeak.gz	H1	None	EZH2
wgEncodeAwgTfbsBroadH1hescJarid1aab26049UniPk.narrowPeak.gz	H1	None	JARID1A
wgEncodeAwgTfbsBroadH1hescRbbp5a300109aUniPk.narrowPeak.gz	H1	None	RBBP5
wgEncodeAwgTfbsBroadHelas3Ezh239875UniPk.narrowPeak.gz	HeLa-S3	None	EZH2
wgEncodeAwgTfbsBroadHelas3Pol2bUniPk.narrowPeak.gz	HeLa-S3	None	Pol2
wgEncodeAwgTfbsBroadHepg2Ezh239875UniPk.narrowPeak.gz	HepG2	None	EZH2
wgEncodeAwgTfbsBroadHmecEzh239875UniPk.narrowPeak.gz	HMEC	None	EZH2
wgEncodeAwgTfbsBroadHsmmEzh239875UniPk.narrowPeak.gz	HSMM	None	EZH2
wgEncodeAwgTfbsBroadHsmmtEzh239875UniPk.narrowPeak.gz	HSMMtube	None	EZH2
wgEncodeAwgTfbsBroadHuvecEzh239875UniPk.narrowPeak.gz	HUVEC	None	EZH2
wgEncodeAwgTfbsBroadHuvecPol2bUniPk.narrowPeak.gz	HUVEC	None	Pol2
wgEncodeAwgTfbsBroadK562Chd1a301218aUniPk.narrowPeak.gz	K562	None	CHD1
wgEncodeAwgTfbsBroadK562Ezh239875UniPk.narrowPeak.gz	K562	None	EZH2
wgEncodeAwgTfbsBroadK562Hdac1sc6298UniPk.narrowPeak.gz	K562	None	HDAC1
wgEncodeAwgTfbsBroadK562Hdac2a300705aUniPk.narrowPeak.gz	K562	None	HDAC2
wgEncodeAwgTfbsBroadK562Hdac6a301341aUniPk.narrowPeak.gz	K562	None	HDAC6
wgEncodeAwgTfbsBroadK562P300UniPk.narrowPeak.gz	K562	None	p300
wgEncodeAwgTfbsBroadK562Phf8a301772aUniPk.narrowPeak.gz	K562	None	PHF8
wgEncodeAwgTfbsBroadK562Plu1UniPk.narrowPeak.gz	K562	None	PLU1
wgEncodeAwgTfbsBroadK562Pol2bUniPk.narrowPeak.gz	K562	None	Pol2
wgEncodeAwgTfbsBroadK562Rbbp5a300109aUniPk.narrowPeak.gz	K562	None	RBBP5
wgEncodeAwgTfbsBroadK562Sap3039731UniPk.narrowPeak.gz	K562	None	SAP30
wgEncodeAwgTfbsBroadNhaEzh239875UniPk.narrowPeak.gz	NH-A	None	EZH2
wgEncodeAwgTfbsBroadNhdfadEzh239875UniPk.narrowPeak.gz	NHDF-Ad	None	EZH2
wgEncodeAwgTfbsBroadNhekEzh239875UniPk.narrowPeak.gz	NHEK	None	EZH2
wgEncodeAwgTfbsBroadNhekPol2bUniPk.narrowPeak.gz	NHEK	None	Pol2
wgEncodeAwgTfbsBroadNhlfEzh239875UniPk.narrowPeak.gz	NHLF	None	EZH2
wgEncodeAwgTfbsHaibGm12878Atf2sc81188V0422111UniPk.narrowPeak.gz	GM12878	None	ATF2
wgEncodeAwgTfbsHaibGm12878Atf3Pcr1xUniPk.narrowPeak.gz	GM12878	None	ATF3
wgEncodeAwgTfbsHaibGm12878BatfPcr1xUniPk.narrowPeak.gz	GM12878	None	BATF
wgEncodeAwgTfbsHaibGm12878Bcl11aPcr1xUniPk.narrowPeak.gz	GM12878	None	BCL11A
wgEncodeAwgTfbsHaibGm12878Bcl3V0416101UniPk.narrowPeak.gz	GM12878	None	BCL3
wgEncodeAwgTfbsHaibGm12878Bclaf101388V0416101UniPk.narrowPeak.gz	GM12878	None	BCLAF1
wgEncodeAwgTfbsHaibGm12878Cebpbsc150V0422111UniPk.narrowPeak.gz	GM12878	None	CEBPB
wgEncodeAwgTfbsHaibGm12878Ebf1sc137065Pcr1xUniPk.narrowPeak.gz	GM12878	None	EBF1
wgEncodeAwgTfbsHaibGm12878Egr1Pcr2xUniPk.narrowPeak.gz	GM12878	None	Egr-1
wgEncodeAwgTfbsHaibGm12878Elf1sc631V0416101UniPk.narrowPeak.gz	GM12878	None	ELF1
wgEncodeAwgTfbsHaibGm12878Ets1Pcr1xUniPk.narrowPeak.gz	GM12878	None	ETS1
wgEncodeAwgTfbsHaibGm12878Foxm1sc502V0422111UniPk.narrowPeak.gz	GM12878	None	FOXM1
wgEncodeAwgTfbsHaibGm12878GabpPcr2xUniPk.narrowPeak.gz	GM12878	None	GABP
wgEncodeAwgTfbsHaibGm12878Irf4sc6059Pcr1xUniPk.narrowPeak.gz	GM12878	None	IRF4
wgEncodeAwgTfbsHaibGm12878Mef2aPcr1xUniPk.narrowPeak.gz	GM12878	None	MEF2A
wgEncodeAwgTfbsHaibGm12878Mef2csc13268V0416101UniPk.narrowPeak.gz	GM12878	None	MEF2C
wgEncodeAwgTfbsHaibGm12878Mta3sc81325V0422111UniPk.narrowPeak.gz	GM12878	None	MTA3
wgEncodeAwgTfbsHaibGm12878Nfatc1sc17834V0422111UniPk.narrowPeak.gz	GM12878	None	NFATC1
wgEncodeAwgTfbsHaibGm12878Nficsc81335V0422111UniPk.narrowPeak.gz	GM12878	None	NFIC
wgEncodeAwgTfbsHaibGm12878NrsfPcr1xUniPk.narrowPeak.gz	GM12878	None	NRSF
wgEncodeAwgTfbsHaibGm12878P300Pcr1xUniPk.narrowPeak.gz	GM12878	None	p300
wgEncodeAwgTfbsHaibGm12878Pax5c20Pcr1xUniPk.narrowPeak.gz	GM12878	None	PAX5
wgEncodeAwgTfbsHaibGm12878Pax5n19Pcr1xUniPk.narrowPeak.gz	GM12878	None	PAX5
wgEncodeAwgTfbsHaibGm12878Pbx3Pcr1xUniPk.narrowPeak.gz	GM12878	None	Pbx3
wgEncodeAwgTfbsHaibGm12878Pmlsc71910V0422111UniPk.narrowPeak.gz	GM12878	None	PML
wgEncodeAwgTfbsHaibGm12878Pol24h8Pcr1xUniPk.narrowPeak.gz	GM12878	None	Pol2
wgEncodeAwgTfbsHaibGm12878Pol2Pcr2xUniPk.narrowPeak.gz	GM12878	None	Pol2
wgEncodeAwgTfbsHaibGm12878Pou2f2Pcr1xUniPk.narrowPeak.gz	GM12878	None	POU2F2
wgEncodeAwgTfbsHaibGm12878Pu1Pcr1xUniPk.narrowPeak.gz	GM12878	None	PU.1
wgEncodeAwgTfbsHaibGm12878Rad21V0416101UniPk.narrowPeak.gz	GM12878	None	Rad21
wgEncodeAwgTfbsHaibGm12878Runx3sc101553V0422111UniPk.narrowPeak.gz	GM12878	None	RUNX3
wgEncodeAwgTfbsHaibGm12878RxraPcr1xUniPk.narrowPeak.gz	GM12878	None	RXRA
wgEncodeAwgTfbsHaibGm12878Six5Pcr1xUniPk.narrowPeak.gz	GM12878	None	SIX5
wgEncodeAwgTfbsHaibGm12878Sp1Pcr1xUniPk.narrowPeak.gz	GM12878	None	SP1
wgEncodeAwgTfbsHaibGm12878SrfPcr2xUniPk.narrowPeak.gz	GM12878	None	SRF
wgEncodeAwgTfbsHaibGm12878Stat5asc74442V0422111UniPk.narrowPeak.gz	GM12878	None	STAT5A
wgEncodeAwgTfbsHaibGm12878Taf1Pcr1xUniPk.narrowPeak.gz	GM12878	None	TAF1
wgEncodeAwgTfbsHaibGm12878Tcf12Pcr1xUniPk.narrowPeak.gz	GM12878	None	TCF12
wgEncodeAwgTfbsHaibGm12878Tcf3Pcr1xUniPk.narrowPeak.gz	GM12878	None	TCF3
wgEncodeAwgTfbsHaibGm12878Usf1Pcr2xUniPk.narrowPeak.gz	GM12878	None	USF-1
wgEncodeAwgTfbsHaibGm12878Yy1sc281Pcr1xUniPk.narrowPeak.gz	GM12878	None	YY1
wgEncodeAwgTfbsHaibGm12878Zbtb33Pcr1xUniPk.narrowPeak.gz	GM12878	None	ZBTB33
wgEncodeAwgTfbsHaibGm12878Zeb1sc25388V0416102UniPk.narrowPeak.gz	GM12878	None	ZEB1
wgEncodeAwgTfbsHaibGm12891Pax5c20V0416101UniPk.narrowPeak.gz	GM12891	None	PAX5
wgEncodeAwgTfbsHaibGm12891Pol24h8Pcr1xUniPk.narrowPeak.gz	GM12891	None	Pol2
wgEncodeAwgTfbsHaibGm12891Pol2Pcr1xUniPk.narrowPeak.gz	GM12891	None	Pol2
wgEncodeAwgTfbsHaibGm12891Pou2f2Pcr1xUniPk.narrowPeak.gz	GM12891	None	POU2F2
wgEncodeAwgTfbsHaibGm12891Pu1Pcr1xUniPk.narrowPeak.gz	GM12891	None	PU.1
wgEncodeAwgTfbsHaibGm12891Taf1Pcr1xUniPk.narrowPeak.gz	GM12891	None	TAF1
wgEncodeAwgTfbsHaibGm12891Yy1sc281V0416101UniPk.narrowPeak.gz	GM12891	None	YY1
wgEncodeAwgTfbsHaibGm12892Pax5c20V0416101UniPk.narrowPeak.gz	GM12892	None	PAX5
wgEncodeAwgTfbsHaibGm12892Pol24h8V0416102UniPk.narrowPeak.gz	GM12892	None	Pol2
wgEncodeAwgTfbsHaibGm12892Pol2V0416102UniPk.narrowPeak.gz	GM12892	None	Pol2
wgEncodeAwgTfbsHaibGm12892Taf1V0416102UniPk.narrowPeak.gz	GM12892	None	TAF1
wgEncodeAwgTfbsHaibGm12892Yy1V0416101UniPk.narrowPeak.gz	GM12892	None	YY1
wgEncodeAwgTfbsHaibH1hescAtf2sc81188V0422111UniPk.narrowPeak.gz	H1	None	ATF2
wgEncodeAwgTfbsHaibH1hescAtf3V0416102UniPk.narrowPeak.gz	H1	None	ATF3
wgEncodeAwgTfbsHaibH1hescBcl11aPcr1xUniPk.narrowPeak.gz	H1	None	BCL11A
wgEncodeAwgTfbsHaibH1hescCtcfsc5916V0416102UniPk.narrowPeak.gz	H1	None	CTCF
wgEncodeAwgTfbsHaibH1hescEgr1V0416102UniPk.narrowPeak.gz	H1	None	Egr-1
wgEncodeAwgTfbsHaibH1hescFosl1sc183V0416102UniPk.narrowPeak.gz	H1	None	FOSL1
wgEncodeAwgTfbsHaibH1hescGabpPcr1xUniPk.narrowPeak.gz	H1	None	GABP
wgEncodeAwgTfbsHaibH1hescHdac2sc6296V0416102UniPk.narrowPeak.gz	H1	None	HDAC2
wgEncodeAwgTfbsHaibH1hescJundV0416102UniPk.narrowPeak.gz	H1	None	JunD
wgEncodeAwgTfbsHaibH1hescNanogsc33759V0416102UniPk.narrowPeak.gz	H1	None	NANOG
wgEncodeAwgTfbsHaibH1hescNrsfV0416102UniPk.narrowPeak.gz	H1	None	NRSF
wgEncodeAwgTfbsHaibH1hescP300V0416102UniPk.narrowPeak.gz	H1	None	p300
wgEncodeAwgTfbsHaibH1hescPol24h8V0416102UniPk.narrowPeak.gz	H1	None	Pol2
wgEncodeAwgTfbsHaibH1hescPol2V0416102UniPk.narrowPeak.gz	H1	None	Pol2
wgEncodeAwgTfbsHaibH1hescPou5f1sc9081V0416102UniPk.narrowPeak.gz	H1	None	POU5F1
wgEncodeAwgTfbsHaibH1hescRad21V0416102UniPk.narrowPeak.gz	H1	None	Rad21
wgEncodeAwgTfbsHaibH1hescRxraV0416102UniPk.narrowPeak.gz	H1	None	RXRA
wgEncodeAwgTfbsHaibH1hescSin3ak20Pcr1xUniPk.narrowPeak.gz	H1	None	Sin3Ak
wgEncodeAwgTfbsHaibH1hescSix5Pcr1xUniPk.narrowPeak.gz	H1	None	SIX5
wgEncodeAwgTfbsHaibH1hescSp1Pcr1xUniPk.narrowPeak.gz	H1	None	SP1
wgEncodeAwgTfbsHaibH1hescSp2V0422111UniPk.narrowPeak.gz	H1	None	SP2
wgEncodeAwgTfbsHaibH1hescSp4v20V0422111UniPk.narrowPeak.gz	H1	None	SP4
wgEncodeAwgTfbsHaibH1hescSrfPcr1xUniPk.narrowPeak.gz	H1	None	SRF
wgEncodeAwgTfbsHaibH1hescTaf1V0416102UniPk.narrowPeak.gz	H1	None	TAF1
wgEncodeAwgTfbsHaibH1hescTaf7sc101167V0416102UniPk.narrowPeak.gz	H1	None	TAF7
wgEncodeAwgTfbsHaibH1hescTcf12Pcr1xUniPk.narrowPeak.gz	H1	None	TCF12
wgEncodeAwgTfbsHaibH1hescTead4sc101184V0422111UniPk.narrowPeak.gz	H1	None	TEAD4
wgEncodeAwgTfbsHaibH1hescUsf1Pcr1xUniPk.narrowPeak.gz	H1	None	USF-1
wgEncodeAwgTfbsHaibH1hescYy1sc281V0416102UniPk.narrowPeak.gz	H1	None	YY1
wgEncodeAwgTfbsHaibHct116Pol24h8V0416101UniPk.narrowPeak.gz	HCT-116	None	Pol2
wgEncodeAwgTfbsHaibHct116Yy1sc281V0416101UniPk.narrowPeak.gz	HCT-116	None	YY1
wgEncodeAwgTfbsHaibHct116Zbtb33V0416101UniPk.narrowPeak.gz	HCT-116	None	ZBTB33
wgEncodeAwgTfbsHaibHelas3GabpPcr1xUniPk.narrowPeak.gz	HeLa-S3	None	GABP
wgEncodeAwgTfbsHaibHelas3NrsfPcr1xUniPk.narrowPeak.gz	HeLa-S3	None	NRSF
wgEncodeAwgTfbsHaibHelas3Pol2Pcr1xUniPk.narrowPeak.gz	HeLa-S3	None	Pol2
wgEncodeAwgTfbsHaibHelas3Taf1Pcr1xUniPk.narrowPeak.gz	HeLa-S3	None	TAF1
wgEncodeAwgTfbsHaibHepg2Atf3V0416101UniPk.narrowPeak.gz	HepG2	None	ATF3
wgEncodeAwgTfbsHaibHepg2Bhlhe40V0416101UniPk.narrowPeak.gz	HepG2	None	BHLHE40
wgEncodeAwgTfbsHaibHepg2Cebpbsc150V0416101UniPk.narrowPeak.gz	HepG2	None	CEBPB
wgEncodeAwgTfbsHaibHepg2Cebpdsc636V0416101UniPk.narrowPeak.gz	HepG2	None	CEBPD
wgEncodeAwgTfbsHaibHepg2Ctcfsc5916V0416101UniPk.narrowPeak.gz	HepG2	None	CTCF
wgEncodeAwgTfbsHaibHepg2Elf1sc631V0416101UniPk.narrowPeak.gz	HepG2	None	ELF1
wgEncodeAwgTfbsHaibHepg2Fosl2V0416101UniPk.narrowPeak.gz	HepG2	None	FOSL2
wgEncodeAwgTfbsHaibHepg2Foxa1sc101058V0416101UniPk.narrowPeak.gz	HepG2	None	FOXA1
wgEncodeAwgTfbsHaibHepg2Foxa1sc6553V0416101UniPk.narrowPeak.gz	HepG2	None	FOXA1
wgEncodeAwgTfbsHaibHepg2Foxa2sc6554V0416101UniPk.narrowPeak.gz	HepG2	None	FOXA2
wgEncodeAwgTfbsHaibHepg2GabpPcr2xUniPk.narrowPeak.gz	HepG2	None	GABP
wgEncodeAwgTfbsHaibHepg2Hdac2sc6296V0416101UniPk.narrowPeak.gz	HepG2	None	HDAC2
wgEncodeAwgTfbsHaibHepg2Hnf4asc8987V0416101UniPk.narrowPeak.gz	HepG2	None	HNF4A
wgEncodeAwgTfbsHaibHepg2Hnf4gsc6558V0416101UniPk.narrowPeak.gz	HepG2	None	HNF4G
wgEncodeAwgTfbsHaibHepg2JundPcr1xUniPk.narrowPeak.gz	HepG2	None	JunD
wgEncodeAwgTfbsHaibHepg2Mbd4sc271530V0422111UniPk.narrowPeak.gz	HepG2	None	MBD4
wgEncodeAwgTfbsHaibHepg2Mybl2sc81192V0422111UniPk.narrowPeak.gz	HepG2	None	MYBL2
wgEncodeAwgTfbsHaibHepg2Nficsc81335V0422111UniPk.narrowPeak.gz	HepG2	None	NFIC
wgEncodeAwgTfbsHaibHepg2NrsfPcr2xUniPk.narrowPeak.gz	HepG2	None	NRSF
wgEncodeAwgTfbsHaibHepg2NrsfV0416101UniPk.narrowPeak.gz	HepG2	None	NRSF
wgEncodeAwgTfbsHaibHepg2P300V0416101UniPk.narrowPeak.gz	HepG2	None	p300
wgEncodeAwgTfbsHaibHepg2Pol24h8V0416102UniPk.narrowPeak.gz	HepG2	None	Pol2
wgEncodeAwgTfbsHaibHepg2Pol2Pcr2xUniPk.narrowPeak.gz	HepG2	None	Pol2
wgEncodeAwgTfbsHaibHepg2Rad21V0416101UniPk.narrowPeak.gz	HepG2	None	Rad21
wgEncodeAwgTfbsHaibHepg2RxraPcr1xUniPk.narrowPeak.gz	HepG2	None	RXRA
wgEncodeAwgTfbsHaibHepg2Sin3ak20Pcr1xUniPk.narrowPeak.gz	HepG2	None	Sin3Ak-20
wgEncodeAwgTfbsHaibHepg2Sp1Pcr1xUniPk.narrowPeak.gz	HepG2	None	SP1
wgEncodeAwgTfbsHaibHepg2Sp2V0422111UniPk.narrowPeak.gz	HepG2	None	SP2
wgEncodeAwgTfbsHaibHepg2SrfV0416101UniPk.narrowPeak.gz	HepG2	None	SRF
wgEncodeAwgTfbsHaibHepg2Taf1Pcr2xUniPk.narrowPeak.gz	HepG2	None	TAF1
wgEncodeAwgTfbsHaibHepg2Tcf12Pcr1xUniPk.narrowPeak.gz	HepG2	None	TCF12
wgEncodeAwgTfbsHaibHepg2Tead4sc101184V0422111UniPk.narrowPeak.gz	HepG2	None	TEAD4
wgEncodeAwgTfbsHaibHepg2Usf1Pcr1xUniPk.narrowPeak.gz	HepG2	None	USF-1
wgEncodeAwgTfbsHaibHepg2Yy1sc281V0416101UniPk.narrowPeak.gz	HepG2	None	YY1
wgEncodeAwgTfbsHaibHepg2Zbtb33Pcr1xUniPk.narrowPeak.gz	HepG2	None	ZBTB33
wgEncodeAwgTfbsHaibHepg2Zbtb7aV0416101UniPk.narrowPeak.gz	HepG2	None	ZBTB7A
wgEncodeAwgTfbsHaibHuvecPol24h8V0416101UniPk.narrowPeak.gz	HUVEC	None	Pol2
wgEncodeAwgTfbsHaibHuvecPol2Pcr1xUniPk.narrowPeak.gz	HUVEC	None	Pol2
wgEncodeAwgTfbsHaibK562Atf3V0416101UniPk.narrowPeak.gz	K562	None	ATF3
wgEncodeAwgTfbsHaibK562Bcl3Pcr1xUniPk.narrowPeak.gz	K562	None	BCL3
wgEncodeAwgTfbsHaibK562Bclaf101388Pcr1xUniPk.narrowPeak.gz	K562	None	BCLAF1
wgEncodeAwgTfbsHaibK562Cbx3sc101004V0422111UniPk.narrowPeak.gz	K562	None	CBX3
wgEncodeAwgTfbsHaibK562Cebpbsc150V0422111UniPk.narrowPeak.gz	K562	None	CEBPB
wgEncodeAwgTfbsHaibK562CtcfcPcr1xUniPk.narrowPeak.gz	K562	None	CTCF
wgEncodeAwgTfbsHaibK562Ctcflsc98982V0416101UniPk.narrowPeak.gz	K562	None	CTCFL
wgEncodeAwgTfbsHaibK562E2f6V0416102UniPk.narrowPeak.gz	K562	None	E2F6
wgEncodeAwgTfbsHaibK562Egr1V0416101UniPk.narrowPeak.gz	K562	None	Egr-1
wgEncodeAwgTfbsHaibK562Elf1sc631V0416102UniPk.narrowPeak.gz	K562	None	ELF1
wgEncodeAwgTfbsHaibK562Ets1V0416101UniPk.narrowPeak.gz	K562	None	ETS1
wgEncodeAwgTfbsHaibK562Fosl1sc183V0416101UniPk.narrowPeak.gz	K562	None	FOSL1
wgEncodeAwgTfbsHaibK562GabpV0416101UniPk.narrowPeak.gz	K562	None	GABP
wgEncodeAwgTfbsHaibK562Gata2sc267Pcr1xUniPk.narrowPeak.gz	K562	None	GATA2
wgEncodeAwgTfbsHaibK562Hdac2sc6296V0416102UniPk.narrowPeak.gz	K562	None	HDAC2
wgEncodeAwgTfbsHaibK562MaxV0416102UniPk.narrowPeak.gz	K562	None	Max
wgEncodeAwgTfbsHaibK562Mef2aV0416101UniPk.narrowPeak.gz	K562	None	MEF2A
wgEncodeAwgTfbsHaibK562Nr2f2sc271940V0422111UniPk.narrowPeak.gz	K562	None	NR2F2
wgEncodeAwgTfbsHaibK562NrsfV0416102UniPk.narrowPeak.gz	K562	None	NRSF
wgEncodeAwgTfbsHaibK562Pmlsc71910V0422111UniPk.narrowPeak.gz	K562	None	PML
wgEncodeAwgTfbsHaibK562Pol24h8V0416101UniPk.narrowPeak.gz	K562	None	Pol2-4H8
wgEncodeAwgTfbsHaibK562Pol2V0416101UniPk.narrowPeak.gz	K562	None	Pol2
wgEncodeAwgTfbsHaibK562Pu1Pcr1xUniPk.narrowPeak.gz	K562	None	PU.1
wgEncodeAwgTfbsHaibK562Rad21V0416102UniPk.narrowPeak.gz	K562	None	Rad21
wgEncodeAwgTfbsHaibK562Sin3ak20V0416101UniPk.narrowPeak.gz	K562	None	Sin3Ak-20
wgEncodeAwgTfbsHaibK562Six5Pcr1xUniPk.narrowPeak.gz	K562	None	SIX5
wgEncodeAwgTfbsHaibK562Sp1Pcr1xUniPk.narrowPeak.gz	K562	None	SP1
wgEncodeAwgTfbsHaibK562Sp2sc643V0416102UniPk.narrowPeak.gz	K562	None	SP2
wgEncodeAwgTfbsHaibK562SrfV0416101UniPk.narrowPeak.gz	K562	None	SRF
wgEncodeAwgTfbsHaibK562Stat5asc74442V0422111UniPk.narrowPeak.gz	K562	None	STAT5A
wgEncodeAwgTfbsHaibK562Taf1V0416101UniPk.narrowPeak.gz	K562	None	TAF1
wgEncodeAwgTfbsHaibK562Taf7sc101167V0416101UniPk.narrowPeak.gz	K562	None	TAF7
wgEncodeAwgTfbsHaibK562Tead4sc101184V0422111UniPk.narrowPeak.gz	K562	None	TEAD4
wgEncodeAwgTfbsHaibK562Thap1sc98174V0416101UniPk.narrowPeak.gz	K562	None	THAP1
wgEncodeAwgTfbsHaibK562Trim28sc81411V0422111UniPk.narrowPeak.gz	K562	None	TRIM28
wgEncodeAwgTfbsHaibK562Usf1V0416101UniPk.narrowPeak.gz	K562	None	USF-1
wgEncodeAwgTfbsHaibK562Yy1V0416101UniPk.narrowPeak.gz	K562	None	YY1
wgEncodeAwgTfbsHaibK562Yy1V0416102UniPk.narrowPeak.gz	K562	None	YY1
wgEncodeAwgTfbsHaibK562Zbtb33Pcr1xUniPk.narrowPeak.gz	K562	None	ZBTB33
wgEncodeAwgTfbsHaibK562Zbtb7asc34508V0416101UniPk.narrowPeak.gz	K562	None	ZBTB7A
wgEncodeAwgTfbsHaibPanc1NrsfPcr2xUniPk.narrowPeak.gz	PANC-1	None	NRSF
wgEncodeAwgTfbsHaibPanc1Pol24h8V0416101UniPk.narrowPeak.gz	PANC-1	None	Pol2-4H8
wgEncodeAwgTfbsHaibPanc1Sin3ak20V0416101UniPk.narrowPeak.gz	PANC-1	None	Sin3Ak-20
wgEncodeAwgTfbsHaibPfsk1Foxp2Pcr2xUniPk.narrowPeak.gz	PFSK-1	None	FOXP2
wgEncodeAwgTfbsHaibPfsk1NrsfPcr2xUniPk.narrowPeak.gz	PFSK-1	None	NRSF
wgEncodeAwgTfbsHaibPfsk1Sin3ak20V0416101UniPk.narrowPeak.gz	PFSK-1	None	Sin3Ak-20
wgEncodeAwgTfbsHaibPfsk1Taf1V0416101UniPk.narrowPeak.gz	PFSK-1	None	TAF1
wgEncodeAwgTfbsHaibSknmcFoxp2Pcr2xUniPk.narrowPeak.gz	SK-N-MC	None	FOXP2
wgEncodeAwgTfbsHaibSknmcPol24h8V0416101UniPk.narrowPeak.gz	SK-N-MC	None	Pol2-4H8
wgEncodeAwgTfbsHaibSknshNrsfPcr2xUniPk.narrowPeak.gz	SK-N-SH	None	NRSF
wgEncodeAwgTfbsHaibSknshNrsfV0416101UniPk.narrowPeak.gz	SK-N-SH	None	NRSF
wgEncodeAwgTfbsHaibSknshPol24h8V0416101UniPk.narrowPeak.gz	SK-N-SH	None	Pol2-4H8
wgEncodeAwgTfbsHaibSknshSin3ak20V0416101UniPk.narrowPeak.gz	SK-N-SH	None	Sin3Ak-20
wgEncodeAwgTfbsHaibSknshTaf1V0416101UniPk.narrowPeak.gz	SK-N-SH	None	TAF1
wgEncodeAwgTfbsHaibSknshraP300V0416102UniPk.narrowPeak.gz	SK-N-SH_RA	None	p300
wgEncodeAwgTfbsHaibSknshraRad21V0416102UniPk.narrowPeak.gz	SK-N-SH_RA	None	Rad21
wgEncodeAwgTfbsHaibSknshraUsf1sc8983V0416102UniPk.narrowPeak.gz	SK-N-SH_RA	None	USF1
wgEncodeAwgTfbsHaibSknshraYy1sc281V0416102UniPk.narrowPeak.gz	SK-N-SH_RA	None	YY1
wgEncodeAwgTfbsHaibU87NrsfPcr2xUniPk.narrowPeak.gz	U87	None	NRSF
wgEncodeAwgTfbsHaibU87Pol24h8V0416101UniPk.narrowPeak.gz	U87	None	Pol2-4H8
wgEncodeAwgTfbsSydhA549Bhlhe40IggrabUniPk.narrowPeak.gz	A549	None	BHLHE40
wgEncodeAwgTfbsSydhA549CebpbIggrabUniPk.narrowPeak.gz	A549	None	CEBPB
wgEncodeAwgTfbsSydhA549MaxIggrabUniPk.narrowPeak.gz	A549	None	Max
wgEncodeAwgTfbsSydhA549Pol2s2IggrabUniPk.narrowPeak.gz	A549	None	Pol2(phosphoS2)
wgEncodeAwgTfbsSydhA549Rad21IggrabUniPk.narrowPeak.gz	A549	None	Rad21
wgEncodeAwgTfbsSydhGm08714Znf274UcdUniPk.narrowPeak.gz	GM08714	None	ZNF274
wgEncodeAwgTfbsSydhGm10847Pol2IggmusUniPk.narrowPeak.gz	GM10847	None	Pol2
wgEncodeAwgTfbsSydhGm12878Bhlhe40cIggmusUniPk.narrowPeak.gz	GM12878	None	BHLHE40
wgEncodeAwgTfbsSydhGm12878Brca1a300IggmusUniPk.narrowPeak.gz	GM12878	None	BRCA1
wgEncodeAwgTfbsSydhGm12878CfosUniPk.narrowPeak.gz	GM12878	None	c-Fos
wgEncodeAwgTfbsSydhGm12878Chd1a301218aIggmusUniPk.narrowPeak.gz	GM12878	None	CHD1
wgEncodeAwgTfbsSydhGm12878Chd2ab68301IggmusUniPk.narrowPeak.gz	GM12878	None	CHD2
wgEncodeAwgTfbsSydhGm12878Corestsc30189IggmusUniPk.narrowPeak.gz	GM12878	None	COREST
wgEncodeAwgTfbsSydhGm12878Ctcfsc15914c20UniPk.narrowPeak.gz	GM12878	None	CTCF
wgEncodeAwgTfbsSydhGm12878E2f4IggmusUniPk.narrowPeak.gz	GM12878	None	E2F4
wgEncodeAwgTfbsSydhGm12878Ebf1sc137065UniPk.narrowPeak.gz	GM12878	None	EBF1
wgEncodeAwgTfbsSydhGm12878Elk112771IggmusUniPk.narrowPeak.gz	GM12878	None	ELK1
wgEncodeAwgTfbsSydhGm12878Ikzf1iknuclaUniPk.narrowPeak.gz	GM12878	None	IKZF1
wgEncodeAwgTfbsSydhGm12878JundUniPk.narrowPeak.gz	GM12878	None	JunD
wgEncodeAwgTfbsSydhGm12878MaxIggmusUniPk.narrowPeak.gz	GM12878	None	Max
wgEncodeAwgTfbsSydhGm12878Mazab85725IggmusUniPk.narrowPeak.gz	GM12878	None	MAZ
wgEncodeAwgTfbsSydhGm12878Mxi1IggmusUniPk.narrowPeak.gz	GM12878	None	Mxi1
wgEncodeAwgTfbsSydhGm12878Nfe2sc22827UniPk.narrowPeak.gz	GM12878	None	NF-E2
wgEncodeAwgTfbsSydhGm12878NfyaIggmusUniPk.narrowPeak.gz	GM12878	None	NF-YA
wgEncodeAwgTfbsSydhGm12878NfybIggmusUniPk.narrowPeak.gz	GM12878	None	NF-YB
wgEncodeAwgTfbsSydhGm12878Nrf1IggmusUniPk.narrowPeak.gz	GM12878	None	Nrf1
wgEncodeAwgTfbsSydhGm12878P300IggmusUniPk.narrowPeak.gz	GM12878	None	p300
wgEncodeAwgTfbsSydhGm12878P300bUniPk.narrowPeak.gz	GM12878	None	p300
wgEncodeAwgTfbsSydhGm12878Pol2IggmusUniPk.narrowPeak.gz	GM12878	None	Pol2
wgEncodeAwgTfbsSydhGm12878Pol2UniPk.narrowPeak.gz	GM12878	None	Pol2
wgEncodeAwgTfbsSydhGm12878Pol2s2IggmusUniPk.narrowPeak.gz	GM12878	None	Pol2(phosphoS2)
wgEncodeAwgTfbsSydhGm12878Pol3UniPk.narrowPeak.gz	GM12878	None	Pol3
wgEncodeAwgTfbsSydhGm12878Rad21IggrabUniPk.narrowPeak.gz	GM12878	None	Rad21
wgEncodeAwgTfbsSydhGm12878Rfx5200401194IggmusUniPk.narrowPeak.gz	GM12878	None	RFX5
wgEncodeAwgTfbsSydhGm12878Sin3anb6001263IggmusUniPk.narrowPeak.gz	GM12878	None	SIN3A
wgEncodeAwgTfbsSydhGm12878Smc3ab9263IggmusUniPk.narrowPeak.gz	GM12878	None	SMC3
wgEncodeAwgTfbsSydhGm12878Stat1UniPk.narrowPeak.gz	GM12878	None	STAT1
wgEncodeAwgTfbsSydhGm12878Stat3IggmusUniPk.narrowPeak.gz	GM12878	None	STAT3
wgEncodeAwgTfbsSydhGm12878Tblr1ab24550IggmusUniPk.narrowPeak.gz	GM12878	None	TBLR1
wgEncodeAwgTfbsSydhGm12878TbpIggmusUniPk.narrowPeak.gz	GM12878	None	TBP
wgEncodeAwgTfbsSydhGm12878Tr4UniPk.narrowPeak.gz	GM12878	None	TR4
wgEncodeAwgTfbsSydhGm12878Usf2IggmusUniPk.narrowPeak.gz	GM12878	None	USF2
wgEncodeAwgTfbsSydhGm12878WhipIggmusUniPk.narrowPeak.gz	GM12878	None	WHIP
wgEncodeAwgTfbsSydhGm12878Yy1UniPk.narrowPeak.gz	GM12878	None	YY1
wgEncodeAwgTfbsSydhGm12878Znf143166181apUniPk.narrowPeak.gz	GM12878	None	Znf143
wgEncodeAwgTfbsSydhGm12878Znf274UniPk.narrowPeak.gz	GM12878	None	ZNF274
wgEncodeAwgTfbsSydhGm12878Zzz3UniPk.narrowPeak.gz	GM12878	None	ZZZ3
wgEncodeAwgTfbsSydhGm12891Pol2IggmusUniPk.narrowPeak.gz	GM12891	None	Pol2
wgEncodeAwgTfbsSydhGm12892Pol2IggmusUniPk.narrowPeak.gz	GM12892	None	Pol2
wgEncodeAwgTfbsSydhGm15510Pol2IggmusUniPk.narrowPeak.gz	GM15510	None	Pol2
wgEncodeAwgTfbsSydhGm18505Pol2IggmusUniPk.narrowPeak.gz	GM18505	None	Pol2
wgEncodeAwgTfbsSydhGm18526Pol2IggmusUniPk.narrowPeak.gz	GM18526	None	Pol2
wgEncodeAwgTfbsSydhGm18951Pol2IggmusUniPk.narrowPeak.gz	GM18951	None	Pol2
wgEncodeAwgTfbsSydhGm19099Pol2IggmusUniPk.narrowPeak.gz	GM19099	None	Pol2
wgEncodeAwgTfbsSydhGm19193Pol2IggmusUniPk.narrowPeak.gz	GM19193	None	Pol2
wgEncodeAwgTfbsSydhH1hescBach1sc14700IggrabUniPk.narrowPeak.gz	H1	None	Bach1
wgEncodeAwgTfbsSydhH1hescBrca1IggrabUniPk.narrowPeak.gz	H1	None	BRCA1
wgEncodeAwgTfbsSydhH1hescCebpbIggrabUniPk.narrowPeak.gz	H1	None	CEBPB
wgEncodeAwgTfbsSydhH1hescChd1a301218aIggrabUniPk.narrowPeak.gz	H1	None	CHD1
wgEncodeAwgTfbsSydhH1hescChd2IggrabUniPk.narrowPeak.gz	H1	None	CHD2
wgEncodeAwgTfbsSydhH1hescCjunIggrabUniPk.narrowPeak.gz	H1	None	c-Jun
wgEncodeAwgTfbsSydhH1hescCmycIggrabUniPk.narrowPeak.gz	H1	None	c-Myc
wgEncodeAwgTfbsSydhH1hescCtbp2UcdUniPk.narrowPeak.gz	H1	None	CtBP2
wgEncodeAwgTfbsSydhH1hescGtf2f1IggrabUniPk.narrowPeak.gz	H1	None	GTF2F1
wgEncodeAwgTfbsSydhH1hescJundIggrabUniPk.narrowPeak.gz	H1	None	JunD
wgEncodeAwgTfbsSydhH1hescMafkIggrabUniPk.narrowPeak.gz	H1	None	MafK
wgEncodeAwgTfbsSydhH1hescMaxUcdUniPk.narrowPeak.gz	H1	None	Max
wgEncodeAwgTfbsSydhH1hescMxi1IggrabUniPk.narrowPeak.gz	H1	None	Mxi1
wgEncodeAwgTfbsSydhH1hescNrf1IggrabUniPk.narrowPeak.gz	H1	None	Nrf1
wgEncodeAwgTfbsSydhH1hescRad21IggrabUniPk.narrowPeak.gz	H1	None	Rad21
wgEncodeAwgTfbsSydhH1hescRfx5200401194IggrabUniPk.narrowPeak.gz	H1	None	RFX5
wgEncodeAwgTfbsSydhH1hescSin3anb6001263IggrabUniPk.narrowPeak.gz	H1	None	SIN3A
wgEncodeAwgTfbsSydhH1hescSuz12UcdUniPk.narrowPeak.gz	H1	None	SUZ12
wgEncodeAwgTfbsSydhH1hescTbpIggrabUniPk.narrowPeak.gz	H1	None	TBP
wgEncodeAwgTfbsSydhH1hescUsf2IggrabUniPk.narrowPeak.gz	H1	None	USF2
wgEncodeAwgTfbsSydhH1hescZnf143IggrabUniPk.narrowPeak.gz	H1	None	Znf143
wgEncodeAwgTfbsSydhHct116Pol2UcdUniPk.narrowPeak.gz	HCT-116	None	Pol2
wgEncodeAwgTfbsSydhHct116Tcf7l2UcdUniPk.narrowPeak.gz	HCT-116	None	TCF7L2
wgEncodeAwgTfbsSydhHek293Elk4UcdUniPk.narrowPeak.gz	HEK293	None	ELK4
wgEncodeAwgTfbsSydhHek293Kap1UcdUniPk.narrowPeak.gz	HEK293	None	KAP1
wgEncodeAwgTfbsSydhHek293Pol2UniPk.narrowPeak.gz	HEK293	None	Pol2
wgEncodeAwgTfbsSydhHek293Tcf7l2UcdUniPk.narrowPeak.gz	HEK293	None	TCF7L2
wgEncodeAwgTfbsSydhHek293tZnf263UcdUniPk.narrowPeak.gz	HEK293-T-REx	None	ZNF263
wgEncodeAwgTfbsSydhHelas3Ap2alphaUniPk.narrowPeak.gz	HeLa-S3	None	AP-2alpha
wgEncodeAwgTfbsSydhHelas3Ap2gammaUniPk.narrowPeak.gz	HeLa-S3	None	AP-2gamma
wgEncodeAwgTfbsSydhHelas3Baf155IggmusUniPk.narrowPeak.gz	HeLa-S3	None	BAF155
wgEncodeAwgTfbsSydhHelas3Baf170IggmusUniPk.narrowPeak.gz	HeLa-S3	None	BAF170
wgEncodeAwgTfbsSydhHelas3Bdp1UniPk.narrowPeak.gz	HeLa-S3	None	BDP1
wgEncodeAwgTfbsSydhHelas3Brca1a300IggrabUniPk.narrowPeak.gz	HeLa-S3	None	BRCA1
wgEncodeAwgTfbsSydhHelas3Brf1UniPk.narrowPeak.gz	HeLa-S3	None	BRF1
wgEncodeAwgTfbsSydhHelas3Brf2UniPk.narrowPeak.gz	HeLa-S3	None	BRF2
wgEncodeAwgTfbsSydhHelas3Brg1IggmusUniPk.narrowPeak.gz	HeLa-S3	None	Brg1
wgEncodeAwgTfbsSydhHelas3CebpbIggrabUniPk.narrowPeak.gz	HeLa-S3	None	CEBPB
wgEncodeAwgTfbsSydhHelas3CfosUniPk.narrowPeak.gz	HeLa-S3	None	c-Fos
wgEncodeAwgTfbsSydhHelas3Chd2IggrabUniPk.narrowPeak.gz	HeLa-S3	None	CHD2
wgEncodeAwgTfbsSydhHelas3CjunIggrabUniPk.narrowPeak.gz	HeLa-S3	None	c-Jun
wgEncodeAwgTfbsSydhHelas3CmycUniPk.narrowPeak.gz	HeLa-S3	None	c-Myc
wgEncodeAwgTfbsSydhHelas3Corestsc30189IggrabUniPk.narrowPeak.gz	HeLa-S3	None	COREST
wgEncodeAwgTfbsSydhHelas3E2f1UniPk.narrowPeak.gz	HeLa-S3	None	E2F1
wgEncodeAwgTfbsSydhHelas3E2f4UniPk.narrowPeak.gz	HeLa-S3	None	E2F4
wgEncodeAwgTfbsSydhHelas3E2f6UniPk.narrowPeak.gz	HeLa-S3	None	E2F6
wgEncodeAwgTfbsSydhHelas3Elk112771IggrabUniPk.narrowPeak.gz	HeLa-S3	None	ELK1
wgEncodeAwgTfbsSydhHelas3Elk4UcdUniPk.narrowPeak.gz	HeLa-S3	None	ELK4
wgEncodeAwgTfbsSydhHelas3Gtf2f1ab28179IggrabUniPk.narrowPeak.gz	HeLa-S3	None	GTF2F1
wgEncodeAwgTfbsSydhHelas3Hae2f1UniPk.narrowPeak.gz	HeLa-S3	None	HA-E2F1
wgEncodeAwgTfbsSydhHelas3Ini1IggmusUniPk.narrowPeak.gz	HeLa-S3	None	Ini1
wgEncodeAwgTfbsSydhHelas3Irf3IggrabUniPk.narrowPeak.gz	HeLa-S3	None	IRF3
wgEncodeAwgTfbsSydhHelas3JundIggrabUniPk.narrowPeak.gz	HeLa-S3	None	JunD
wgEncodeAwgTfbsSydhHelas3MafkIggrabUniPk.narrowPeak.gz	HeLa-S3	None	MafK
wgEncodeAwgTfbsSydhHelas3MaxIggrabUniPk.narrowPeak.gz	HeLa-S3	None	Max
wgEncodeAwgTfbsSydhHelas3Mazab85725IggrabUniPk.narrowPeak.gz	HeLa-S3	None	MAZ
wgEncodeAwgTfbsSydhHelas3Mxi1af4185IggrabUniPk.narrowPeak.gz	HeLa-S3	None	Mxi1
wgEncodeAwgTfbsSydhHelas3NfyaIggrabUniPk.narrowPeak.gz	HeLa-S3	None	NF-YA
wgEncodeAwgTfbsSydhHelas3NfybIggrabUniPk.narrowPeak.gz	HeLa-S3	None	NF-YB
wgEncodeAwgTfbsSydhHelas3Nrf1IggmusUniPk.narrowPeak.gz	HeLa-S3	None	Nrf1
wgEncodeAwgTfbsSydhHelas3P300sc584sc584IggrabUniPk.narrowPeak.gz	HeLa-S3	None	p300
wgEncodeAwgTfbsSydhHelas3Pol2UniPk.narrowPeak.gz	HeLa-S3	None	Pol2
wgEncodeAwgTfbsSydhHelas3Pol2s2IggrabUniPk.narrowPeak.gz	HeLa-S3	None	Pol2(phosphoS2)
wgEncodeAwgTfbsSydhHelas3Prdm19115IggrabUniPk.narrowPeak.gz	HeLa-S3	None	PRDM1
wgEncodeAwgTfbsSydhHelas3Rad21IggrabUniPk.narrowPeak.gz	HeLa-S3	None	Rad21
wgEncodeAwgTfbsSydhHelas3Rfx5200401194IggrabUniPk.narrowPeak.gz	HeLa-S3	None	RFX5
wgEncodeAwgTfbsSydhHelas3Rpc155UniPk.narrowPeak.gz	HeLa-S3	None	RPC155
wgEncodeAwgTfbsSydhHelas3Smc3ab9263IggrabUniPk.narrowPeak.gz	HeLa-S3	None	SMC3
wgEncodeAwgTfbsSydhHelas3Spt20UniPk.narrowPeak.gz	HeLa-S3	None	SPT20
wgEncodeAwgTfbsSydhHelas3Stat3IggrabUniPk.narrowPeak.gz	HeLa-S3	None	STAT3
wgEncodeAwgTfbsSydhHelas3TbpIggrabUniPk.narrowPeak.gz	HeLa-S3	None	TBP
wgEncodeAwgTfbsSydhHelas3Tcf7l2UcdUniPk.narrowPeak.gz	HeLa-S3	None	TCF7L2
wgEncodeAwgTfbsSydhHelas3Tcf7l2c9b92565UcdUniPk.narrowPeak.gz	HeLa-S3	None	TCF7L2_C9B9
wgEncodeAwgTfbsSydhHelas3Tf3c110UniPk.narrowPeak.gz	HeLa-S3	None	TFIIIC-110
wgEncodeAwgTfbsSydhHelas3Tr4UniPk.narrowPeak.gz	HeLa-S3	None	TR4
wgEncodeAwgTfbsSydhHelas3Usf2IggmusUniPk.narrowPeak.gz	HeLa-S3	None	USF2
wgEncodeAwgTfbsSydhHelas3Zkscan1hpa006672IggrabUniPk.narrowPeak.gz	HeLa-S3	None	ZKSCAN1
wgEncodeAwgTfbsSydhHelas3Znf143IggrabUniPk.narrowPeak.gz	HeLa-S3	None	Znf143
wgEncodeAwgTfbsSydhHelas3Znf274UcdUniPk.narrowPeak.gz	HeLa-S3	None	ZNF274
wgEncodeAwgTfbsSydhHelas3Zzz3UniPk.narrowPeak.gz	HeLa-S3	None	ZZZ3
wgEncodeAwgTfbsSydhHepg2Arid3anb100279IggrabUniPk.narrowPeak.gz	HepG2	None	ARID3A
wgEncodeAwgTfbsSydhHepg2Bhlhe40cIggrabUniPk.narrowPeak.gz	HepG2	None	BHLHE40
wgEncodeAwgTfbsSydhHepg2Brca1a300IggrabUniPk.narrowPeak.gz	HepG2	None	BRCA1
wgEncodeAwgTfbsSydhHepg2CebpbIggrabUniPk.narrowPeak.gz	HepG2	None	CEBPB
wgEncodeAwgTfbsSydhHepg2Chd2ab68301IggrabUniPk.narrowPeak.gz	HepG2	None	CHD2
wgEncodeAwgTfbsSydhHepg2CjunIggrabUniPk.narrowPeak.gz	HepG2	None	c-Jun
wgEncodeAwgTfbsSydhHepg2Corestsc30189IggrabUniPk.narrowPeak.gz	HepG2	None	COREST
wgEncodeAwgTfbsSydhHepg2Irf3IggrabUniPk.narrowPeak.gz	HepG2	None	IRF3
wgEncodeAwgTfbsSydhHepg2JundIggrabUniPk.narrowPeak.gz	HepG2	None	JunD
wgEncodeAwgTfbsSydhHepg2Maffm8194IggrabUniPk.narrowPeak.gz	HepG2	None	MafF
wgEncodeAwgTfbsSydhHepg2Mafkab50322IggrabUniPk.narrowPeak.gz	HepG2	None	MafK
wgEncodeAwgTfbsSydhHepg2Mafksc477IggrabUniPk.narrowPeak.gz	HepG2	None	MafK
wgEncodeAwgTfbsSydhHepg2MaxIggrabUniPk.narrowPeak.gz	HepG2	None	Max
wgEncodeAwgTfbsSydhHepg2Mazab85725IggrabUniPk.narrowPeak.gz	HepG2	None	MAZ
wgEncodeAwgTfbsSydhHepg2Mxi1UniPk.narrowPeak.gz	HepG2	None	Mxi1
wgEncodeAwgTfbsSydhHepg2Nrf1IggrabUniPk.narrowPeak.gz	HepG2	None	Nrf1
wgEncodeAwgTfbsSydhHepg2P300sc582IggrabUniPk.narrowPeak.gz	HepG2	None	p300
wgEncodeAwgTfbsSydhHepg2Pol2IggrabUniPk.narrowPeak.gz	HepG2	None	Pol2
wgEncodeAwgTfbsSydhHepg2Pol2s2IggrabUniPk.narrowPeak.gz	HepG2	None	Pol2(phosphoS2)
wgEncodeAwgTfbsSydhHepg2Rad21IggrabUniPk.narrowPeak.gz	HepG2	None	Rad21
wgEncodeAwgTfbsSydhHepg2Rfx5200401194IggrabUniPk.narrowPeak.gz	HepG2	None	RFX5
wgEncodeAwgTfbsSydhHepg2Smc3ab9263IggrabUniPk.narrowPeak.gz	HepG2	None	SMC3
wgEncodeAwgTfbsSydhHepg2TbpIggrabUniPk.narrowPeak.gz	HepG2	None	TBP
wgEncodeAwgTfbsSydhHepg2Tcf7l2UcdUniPk.narrowPeak.gz	HepG2	None	TCF7L2
wgEncodeAwgTfbsSydhHepg2Tr4UcdUniPk.narrowPeak.gz	HepG2	None	TR4
wgEncodeAwgTfbsSydhHepg2Usf2IggrabUniPk.narrowPeak.gz	HepG2	None	USF2
wgEncodeAwgTfbsSydhHepg2Znf274UcdUniPk.narrowPeak.gz	HepG2	None	ZNF274
wgEncodeAwgTfbsSydhHuvecCfosUcdUniPk.narrowPeak.gz	HUVEC	None	c-Fos
wgEncodeAwgTfbsSydhHuvecCjunUniPk.narrowPeak.gz	HUVEC	None	c-Jun
wgEncodeAwgTfbsSydhHuvecGata2UcdUniPk.narrowPeak.gz	HUVEC	None	GATA-2
wgEncodeAwgTfbsSydhHuvecMaxUniPk.narrowPeak.gz	HUVEC	None	Max
wgEncodeAwgTfbsSydhHuvecPol2UniPk.narrowPeak.gz	HUVEC	None	Pol2
wgEncodeAwgTfbsSydhImr90CebpbIggrabUniPk.narrowPeak.gz	IMR90	None	CEBPB
wgEncodeAwgTfbsSydhImr90CtcfbIggrabUniPk.narrowPeak.gz	IMR90	None	CTCF
wgEncodeAwgTfbsSydhImr90MafkIggrabUniPk.narrowPeak.gz	IMR90	None	MafK
wgEncodeAwgTfbsSydhImr90Pol2IggrabUniPk.narrowPeak.gz	IMR90	None	Pol2
wgEncodeAwgTfbsSydhImr90Rad21IggrabUniPk.narrowPeak.gz	IMR90	None	Rad21
wgEncodeAwgTfbsSydhK562Arid3asc8821IggrabUniPk.narrowPeak.gz	K562	None	ARID3A
wgEncodeAwgTfbsSydhK562Atf106325UniPk.narrowPeak.gz	K562	None	ATF1
wgEncodeAwgTfbsSydhK562Atf3UniPk.narrowPeak.gz	K562	None	ATF3
wgEncodeAwgTfbsSydhK562Bach1sc14700IggrabUniPk.narrowPeak.gz	K562	None	Bach1
wgEncodeAwgTfbsSydhK562Bdp1UniPk.narrowPeak.gz	K562	None	BDP1
wgEncodeAwgTfbsSydhK562Bhlhe40nb100IggrabUniPk.narrowPeak.gz	K562	None	BHLHE40
wgEncodeAwgTfbsSydhK562Brf1UniPk.narrowPeak.gz	K562	None	BRF1
wgEncodeAwgTfbsSydhK562Brf2UniPk.narrowPeak.gz	K562	None	BRF2
wgEncodeAwgTfbsSydhK562Brg1IggmusUniPk.narrowPeak.gz	K562	None	Brg1
wgEncodeAwgTfbsSydhK562Ccnt2UniPk.narrowPeak.gz	K562	None	CCNT2
wgEncodeAwgTfbsSydhK562CebpbIggrabUniPk.narrowPeak.gz	K562	None	CEBPB
wgEncodeAwgTfbsSydhK562CfosUniPk.narrowPeak.gz	K562	None	c-Fos
wgEncodeAwgTfbsSydhK562Chd2ab68301IggrabUniPk.narrowPeak.gz	K562	None	CHD2
wgEncodeAwgTfbsSydhK562CjunUniPk.narrowPeak.gz	K562	None	c-Jun
wgEncodeAwgTfbsSydhK562CmycIggrabUniPk.narrowPeak.gz	K562	None	c-Myc
wgEncodeAwgTfbsSydhK562CmycUniPk.narrowPeak.gz	K562	None	c-Myc
wgEncodeAwgTfbsSydhK562Corestab24166IggrabUniPk.narrowPeak.gz	K562	None	COREST
wgEncodeAwgTfbsSydhK562Corestsc30189IggrabUniPk.narrowPeak.gz	K562	None	COREST
wgEncodeAwgTfbsSydhK562CtcfbIggrabUniPk.narrowPeak.gz	K562	None	CTCF
wgEncodeAwgTfbsSydhK562E2f4UcdUniPk.narrowPeak.gz	K562	None	E2F4
wgEncodeAwgTfbsSydhK562E2f6UcdUniPk.narrowPeak.gz	K562	None	E2F6
wgEncodeAwgTfbsSydhK562Elk112771IggrabUniPk.narrowPeak.gz	K562	None	ELK1
wgEncodeAwgTfbsSydhK562Gata1UcdUniPk.narrowPeak.gz	K562	None	GATA-1
wgEncodeAwgTfbsSydhK562Gata2UcdUniPk.narrowPeak.gz	K562	None	GATA-2
wgEncodeAwgTfbsSydhK562Gtf2bUniPk.narrowPeak.gz	K562	None	GTF2B
wgEncodeAwgTfbsSydhK562Gtf2f1ab28179IggrabUniPk.narrowPeak.gz	K562	None	GTF2F1
wgEncodeAwgTfbsSydhK562Hmgn3UniPk.narrowPeak.gz	K562	None	HMGN3
wgEncodeAwgTfbsSydhK562Ini1IggmusUniPk.narrowPeak.gz	K562	None	Ini1
wgEncodeAwgTfbsSydhK562JundIggrabUniPk.narrowPeak.gz	K562	None	JunD
wgEncodeAwgTfbsSydhK562Kap1UcdUniPk.narrowPeak.gz	K562	None	KAP1
wgEncodeAwgTfbsSydhK562MaffIggrabUniPk.narrowPeak.gz	K562	None	MafF
wgEncodeAwgTfbsSydhK562Mafkab50322IggrabUniPk.narrowPeak.gz	K562	None	MafK
wgEncodeAwgTfbsSydhK562MaxIggrabUniPk.narrowPeak.gz	K562	None	Max
wgEncodeAwgTfbsSydhK562Mazab85725IggrabUniPk.narrowPeak.gz	K562	None	MAZ
wgEncodeAwgTfbsSydhK562Mxi1af4185IggrabUniPk.narrowPeak.gz	K562	None	Mxi1
wgEncodeAwgTfbsSydhK562NelfeUniPk.narrowPeak.gz	K562	None	NELFe
wgEncodeAwgTfbsSydhK562Nfe2UniPk.narrowPeak.gz	K562	None	NF-E2
wgEncodeAwgTfbsSydhK562NfyaUniPk.narrowPeak.gz	K562	None	NF-YA
wgEncodeAwgTfbsSydhK562NfybUniPk.narrowPeak.gz	K562	None	NF-YB
wgEncodeAwgTfbsSydhK562Nrf1IggrabUniPk.narrowPeak.gz	K562	None	Nrf1
wgEncodeAwgTfbsSydhK562P300IggrabUniPk.narrowPeak.gz	K562	None	p300
wgEncodeAwgTfbsSydhK562Pol2IggmusUniPk.narrowPeak.gz	K562	None	Pol2
wgEncodeAwgTfbsSydhK562Pol2UniPk.narrowPeak.gz	K562	None	Pol2
wgEncodeAwgTfbsSydhK562Pol2s2IggrabUniPk.narrowPeak.gz	K562	None	Pol2(phosphoS2)
wgEncodeAwgTfbsSydhK562Pol2s2UniPk.narrowPeak.gz	K562	None	Pol2(phosphoS2)
wgEncodeAwgTfbsSydhK562Pol3UniPk.narrowPeak.gz	K562	None	Pol3
wgEncodeAwgTfbsSydhK562Rad21UniPk.narrowPeak.gz	K562	None	Rad21
wgEncodeAwgTfbsSydhK562Rfx5IggrabUniPk.narrowPeak.gz	K562	None	RFX5
wgEncodeAwgTfbsSydhK562Rpc155UniPk.narrowPeak.gz	K562	None	RPC155
wgEncodeAwgTfbsSydhK562Setdb1UcdUniPk.narrowPeak.gz	K562	None	SETDB1
wgEncodeAwgTfbsSydhK562Sirt6UniPk.narrowPeak.gz	K562	None	SIRT6
wgEncodeAwgTfbsSydhK562Smc3ab9263IggrabUniPk.narrowPeak.gz	K562	None	SMC3
wgEncodeAwgTfbsSydhK562Tal1sc12984IggmusUniPk.narrowPeak.gz	K562	None	TAL1
wgEncodeAwgTfbsSydhK562Tblr1ab24550IggrabUniPk.narrowPeak.gz	K562	None	TBLR1
wgEncodeAwgTfbsSydhK562Tblr1nb600270IggrabUniPk.narrowPeak.gz	K562	None	TBLR1
wgEncodeAwgTfbsSydhK562TbpIggmusUniPk.narrowPeak.gz	K562	None	TBP
wgEncodeAwgTfbsSydhK562Tf3c110UniPk.narrowPeak.gz	K562	None	TFIIIC-110
wgEncodeAwgTfbsSydhK562Tr4UcdUniPk.narrowPeak.gz	K562	None	TR4
wgEncodeAwgTfbsSydhK562Ubfsc13125IggmusUniPk.narrowPeak.gz	K562	None	UBF
wgEncodeAwgTfbsSydhK562Ubtfsab1404509IggmusUniPk.narrowPeak.gz	K562	None	UBTF
wgEncodeAwgTfbsSydhK562Usf2IggrabUniPk.narrowPeak.gz	K562	None	USF2
wgEncodeAwgTfbsSydhK562Yy1UcdUniPk.narrowPeak.gz	K562	None	YY1
wgEncodeAwgTfbsSydhK562Znf143IggrabUniPk.narrowPeak.gz	K562	None	Znf143
wgEncodeAwgTfbsSydhK562Znf263UcdUniPk.narrowPeak.gz	K562	None	ZNF263
wgEncodeAwgTfbsSydhK562Znf274UcdUniPk.narrowPeak.gz	K562	None	ZNF274
wgEncodeAwgTfbsSydhK562Znf274m01UcdUniPk.narrowPeak.gz	K562	None	ZNF274
wgEncodeAwgTfbsSydhMcf7Gata3UcdUniPk.narrowPeak.gz	MCF-7	None	GATA3
wgEncodeAwgTfbsSydhMcf7Gata3sc269UcdUniPk.narrowPeak.gz	MCF-7	None	GATA3
wgEncodeAwgTfbsSydhMcf7Hae2f1UcdUniPk.narrowPeak.gz	MCF-7	None	HA-E2F1
wgEncodeAwgTfbsSydhMcf7Tcf7l2UcdUniPk.narrowPeak.gz	MCF-7	None	TCF7L2
wgEncodeAwgTfbsSydhMcf7Znf217UcdUniPk.narrowPeak.gz	MCF-7	None	ZNF217
wgEncodeAwgTfbsSydhNb4CmycUniPk.narrowPeak.gz	NB4	None	c-Myc
wgEncodeAwgTfbsSydhNb4MaxUniPk.narrowPeak.gz	NB4	None	Max
wgEncodeAwgTfbsSydhNb4Pol2UniPk.narrowPeak.gz	NB4	None	Pol2
wgEncodeAwgTfbsSydhNt2d1Suz12UcdUniPk.narrowPeak.gz	NT2-D1	None	SUZ12
wgEncodeAwgTfbsSydhNt2d1Yy1UcdUniPk.narrowPeak.gz	NT2-D1	None	YY1
wgEncodeAwgTfbsSydhNt2d1Znf274UcdUniPk.narrowPeak.gz	NT2-D1	None	ZNF274
wgEncodeAwgTfbsSydhPanc1Tcf7l2UcdUniPk.narrowPeak.gz	PANC-1	None	TCF7L2
wgEncodeAwgTfbsSydhPbdeGata1UcdUniPk.narrowPeak.gz	PBDE	None	GATA-1
wgEncodeAwgTfbsSydhPbdePol2UcdUniPk.narrowPeak.gz	PBDE	None	Pol2
wgEncodeAwgTfbsSydhPbdefetalGata1UcdUniPk.narrowPeak.gz	PBDEFetal	None	GATA-1
wgEncodeAwgTfbsSydhRajiPol2UcdUniPk.narrowPeak.gz	Raji	None	Pol2
wgEncodeAwgTfbsSydhShsy5yGata2UcdUniPk.narrowPeak.gz	SH-SY5Y	None	GATA-2
wgEncodeAwgTfbsSydhShsy5yGata3sc269sc269UcdUniPk.narrowPeak.gz	SH-SY5Y	None	GATA3
wgEncodeAwgTfbsSydhU2osKap1UcdUniPk.narrowPeak.gz	U2OS	None	KAP1
wgEncodeAwgTfbsSydhU2osSetdb1UcdUniPk.narrowPeak.gz	U2OS	None	SETDB1
wgEncodeAwgTfbsUchicagoK562EfosUniPk.narrowPeak.gz	K562	None	FOS
wgEncodeAwgTfbsUchicagoK562Egata2UniPk.narrowPeak.gz	K562	None	GATA2
wgEncodeAwgTfbsUchicagoK562Ehdac8UniPk.narrowPeak.gz	K562	None	HDAC8
wgEncodeAwgTfbsUchicagoK562EjunbUniPk.narrowPeak.gz	K562	None	JunB
wgEncodeAwgTfbsUchicagoK562EjundUniPk.narrowPeak.gz	K562	None	JunD
wgEncodeAwgTfbsUtaA549Pol2UniPk.narrowPeak.gz	A549	None	Pol2
wgEncodeAwgTfbsUtaGlioblaPol2UniPk.narrowPeak.gz	Gliobla	None	Pol2
wgEncodeAwgTfbsUtaGm12878CmycUniPk.narrowPeak.gz	GM12878	None	c-Myc
wgEncodeAwgTfbsUtaGm12878Pol2UniPk.narrowPeak.gz	GM12878	None	Pol2
wgEncodeAwgTfbsUtaH1hescCmycUniPk.narrowPeak.gz	H1	None	c-Myc
wgEncodeAwgTfbsUtaH1hescPol2UniPk.narrowPeak.gz	H1	None	Pol2
wgEncodeAwgTfbsUtaHelas3CmycUniPk.narrowPeak.gz	HeLa-S3	None	c-Myc
wgEncodeAwgTfbsUtaHelas3Pol2UniPk.narrowPeak.gz	HeLa-S3	None	Pol2
wgEncodeAwgTfbsUtaHepg2CmycUniPk.narrowPeak.gz	HepG2	None	c-Myc
wgEncodeAwgTfbsUtaHepg2Pol2UniPk.narrowPeak.gz	HepG2	None	Pol2
wgEncodeAwgTfbsUtaHuvecCmycUniPk.narrowPeak.gz	HUVEC	None	c-Myc
wgEncodeAwgTfbsUtaHuvecPol2UniPk.narrowPeak.gz	HUVEC	None	Pol2
wgEncodeAwgTfbsUtaK562CmycUniPk.narrowPeak.gz	K562	None	c-Myc
wgEncodeAwgTfbsUtaK562Pol2UniPk.narrowPeak.gz	K562	None	Pol2
wgEncodeAwgTfbsUtaMcf7Pol2UniPk.narrowPeak.gz	MCF-7	None	Pol2
wgEncodeAwgTfbsUtaProgfibPol2UniPk.narrowPeak.gz	ProgFib	None	Pol2

## Sheet: S6

Table S6 miRNA families to run targetScan prediction
miRNA family	Seed
let-7/98/4458/4500	GAGGUAG
miR-101/101ab	ACAGUAC
miR-103a/107/107ab	GCAGCAU
miR-10abc/10a-5p	ACCCUGU
miR-122/122a/1352	GGAGUGU
miR-124/124ab/506	AAGGCAC
miR-125a-5p/125b-5p/351/670/4319	CCCUGAG
miR-126-3p	CGUACCG
miR-128/128ab	CACAGUG
miR-129-5p/129ab-5p	UUUUUGC
miR-130ac/301ab/301b/301b-3p/454/721/4295/3666	AGUGCAA
miR-132/212/212-3p	AACAGUC
miR-133abc	UUGGUCC
miR-135ab/135a-5p	AUGGCUU
miR-137/137ab	UAUUGCU
miR-138/138ab	GCUGGUG
miR-139-5p	CUACAGU
miR-140/140-5p/876-3p/1244	AGUGGUU
miR-141/200a	AACACUG
miR-142-3p	GUAGUGU
miR-143/1721/4770	GAGAUGA
miR-144	ACAGUAU
miR-145	UCCAGUU
miR-146ac/146b-5p	GAGAACU
miR-148ab-3p/152	CAGUGCA
miR-150/5127	CUCCCAA
miR-153	UGCAUAG
miR-155	UAAUGCU
miR-15abc/16/16abc/195/322/424/497/1907	AGCAGCA
miR-17/17-5p/20ab/20b-5p/93/106ab/427/518a-3p/519d	AAAGUGC
miR-181abcd/4262	ACAUUCA
miR-182	UUGGCAA
miR-183	AUGGCAC
miR-184	GGACGGA
miR-187	CGUGUCU
miR-18ab/4735-3p	AAGGUGC
miR-190/190ab	GAUAUGU
miR-191	AACGGAA
miR-192/215	UGACCUA
miR-193/193b/193a-3p	ACUGGCC
miR-194	GUAACAG
miR-196abc	AGGUAGU
miR-199ab-5p	CCAGUGU
miR-19ab	GUGCAAA
miR-1ab/206/613	GGAAUGU
miR-200bc/429/548a	AAUACUG
miR-203	UGAAAUG
miR-204/204b/211	UCCCUUU
miR-205/205ab	CCUUCAU
miR-208ab/208ab-3p	UAAGACG
miR-21/590-5p	AGCUUAU
miR-210	UGUGCGU
miR-214/761/3619-5p	CAGCAGG
miR-216a	AAUCUCA
miR-216b/216b-5p	AAUCUCU
miR-217	ACUGCAU
miR-218/218a	UGUGCUU
miR-219-5p/508/508-3p/4782-3p	GAUUGUC
miR-22/22-3p	AGCUGCC
miR-221/222/222ab/1928	GCUACAU
miR-223	GUCAGUU
miR-23abc/23b-3p	UCACAUU
miR-24/24ab/24-3p	GGCUCAG
miR-25/32/92abc/363/363-3p/367	AUUGCAC
miR-26ab/1297/4465	UCAAGUA
miR-27abc/27a-3p	UCACAGU
miR-29abcd	AGCACCA
miR-30abcdef/30abe-5p/384-5p	GUAAACA
miR-31	GGCAAGA
miR-338/338-3p	CCAGCAU
miR-33a-3p/365/365-3p	AAUGCCC
miR-33ab/33-5p	UGCAUUG
miR-34ac/34bc-5p/449abc/449c-5p	GGCAGUG
miR-375	UUGUUCG
miR-383	GAUCAGA
miR-425/425-5p/489	AUGACAC
miR-451	AACCGUU
miR-455-5p	AUGUGCC
miR-490-3p	AACCUGG
miR-499-5p	UAAGACU
miR-503	AGCAGCG
miR-551a	CGACCCA
miR-7/7ab	GGAAGAC
miR-9/9ab	CUUUGGU
miR-93/93a/105/106a/291a-3p/294/295/302abcde/372/373/428/519a/520be/520acd-3p/1378/1420ac	AAGUGCU
miR-96/507/1271	UUGGCAC
miR-99ab/100	ACCCGUA

## Sheet: S7

Table S7 CLIP-Seq datasets of AGO
Series	Sample	CLIP-Seq type	# Mapped reads	# Unique mapped reads	# Cross-linking sites
GSE41272	GSM1013104	HITS-CLIP	1879737	1738182	3811
GSE41272	GSM1013105	HITS-CLIP	8205516	6949943	13615
GSE41272	GSM1013106	HITS-CLIP	5438292	4514435	6546
GSE41272	GSM1013107	HITS-CLIP	931770	720283	2519
GSE41272	GSM1013109	HITS-CLIP	1385585	1224233	2135
GSE41272	GSM1013110	HITS-CLIP	794972	693838	2626
GSE41272	GSM1013111	HITS-CLIP	2530901	1800289	3275
GSE41272	GSM1013112	HITS-CLIP	2451006	1964942	1336
GSE41272	GSM1013113	HITS-CLIP	2050911	1760320	749
GSE41272	GSM1013114	HITS-CLIP	1327648	928913	1276
GSE41272	GSM1013115	HITS-CLIP	2717201	2334004	844
GSE41272	GSM1013116	HITS-CLIP	1710379	1219937	1314
GSE41272	GSM1013117	HITS-CLIP	2192641	1862365	680
GSE41272	GSM1013118	HITS-CLIP	1297596	948339	789
GSE41357	GSM1015448	HITS-CLIP	66656638	28977601	17422
GSE41357	GSM1015449	HITS-CLIP	34712736	21294892	12339
GSE41437	GSM1020022	PAR-CLIP	195094	70048	62
GSE41437	GSM1020023	PAR-CLIP	117347	47592	11
GSE41437	GSM1020025	PAR-CLIP	140121	65681	9
GSE42701	GSM1048187	HITS-CLIP	24324060	16626279	35617
GSE42701	GSM1048188	HITS-CLIP	21905569	13576144	41159
GSE43573	GSM1065667	PAR-CLIP	10070351	4588548	27378
GSE43573	GSM1065668	PAR-CLIP	1742723	913203	1643
GSE43573	GSM1065669	PAR-CLIP	2604481	1047948	6577
GSE43573	GSM1065670	PAR-CLIP	5688461	2981289	10441
GSE43909	GSM1074234	PAR-CLIP	510492	265805	1461
GSE44376	GSM1084056	HITS-CLIP	172558	58709	385
GSE44376	GSM1084057	HITS-CLIP	195325	63892	646
GSE44377	GSM1084067	HITS-CLIP	1447132	609405	935
GSE44377	GSM1084068	HITS-CLIP	1035422	396930	1815
GSE44377	GSM1084069	HITS-CLIP	1172549	449993	2110
GSE44378	GSM1084072	HITS-CLIP	224661	94083	308
GSE44378	GSM1084073	HITS-CLIP	229180	91538	356
GSE44378	GSM1084076	HITS-CLIP	286788	121279	304
GSE44378	GSM1084077	HITS-CLIP	268109	113910	265
GSE44378	GSM1084078	HITS-CLIP	1517510	564360	550
GSE44378	GSM1084080	HITS-CLIP	2061761	791330	385
GSE44378	GSM1084081	HITS-CLIP	2003146	824102	796
GSE46611	GSM1133252	PAR-CLIP	158457	64773	9
GSE51924	GSM1255336	HITS-CLIP	3816594	3293574	5327
GSE52082	GSM1259105	HITS-CLIP	1153559	243494	9319
GSE52082	GSM1259113	HITS-CLIP	1018662	204160	8320
GSE52082	GSM1259114	HITS-CLIP	6168606	825818	6856
GSE52082	GSM1259115	HITS-CLIP	2230458	436672	8953
GSE52083	GSM1259118	HITS-CLIP	48755	13944	536
GSE56836	GSM1370382	PAR-CLIP	6263463	1791689	222
GSE56836	GSM1370383	PAR-CLIP	11196951	2864108	391
GSE56836	GSM1370384	PAR-CLIP	10485698	2649419	382
GSE56836	GSM1370385	PAR-CLIP	7628116	2162847	444
GSE21918	GSM545212	PAR-CLIP	1655477	585799	4710
GSE21918	GSM545213	PAR-CLIP	1979728	546299	559
GSE21918	GSM545214	PAR-CLIP	3466975	1259964	9080
GSE21918	GSM545215	PAR-CLIP	3014072	899450	963
GSE21918	GSM545216	PAR-CLIP	2618469	1188784	13808
GSE21918	GSM545217	PAR-CLIP	1680797	447867	3461
GSE28859	GSM714642	HITS-CLIP	17384994	8952729	30062
GSE28859	GSM714643	HITS-CLIP	3839178	1575304	3805
GSE28859	GSM714644	PAR-CLIP	12254115	5810906	33642
GSE28859	GSM714645	PAR-CLIP	3275399	1417241	6105
GSE28859	GSM714646	PAR-CLIP	28326599	14747332	5608
GSE28859	GSM714647	PAR-CLIP	21905010	10794856	3326

## Sheet: S8

Table S8 CLIP-Seq datasets of RNA binding proteins
SRA025082	Sample	RBP	Cell line	Treatment	CLIP-Seq type	# Mapped reads	# Unique mapped reads	# Cross-linking sites
GSE40778	GSM1001330	EIF4A3	HeLa	None	HITS-CLIP	771792	635902	13821
GSE40778	GSM1001331	EIF4A3	HeLa	None	HITS-CLIP	5298064	3710138	48656
GSE42701	GSM1048186	PTBP1	Hela	None	HITS-CLIP	2784651	1765826	30217
GSE42701	GSM1048189	PTBP1	Hela	treated with control shRNA	HITS-CLIP	35242809	30155987	128260
GSE42701	GSM1048190	PTBP1	Hela	treated with PTB shRNA	HITS-CLIP	34058660	29059223	135155
GSE42701	GSM1048191	PTBP1	Hela	treated with control shRNA and further treated with actinomycin D (2 ug/ml) for 4 hours	HITS-CLIP	6922824	5226416	10247
GSE42701	GSM1048192	PTBP1	Hela	treated with PTB shRNA and further treated with actinomycin D (2 ug/ml) for 4 hours	HITS-CLIP	4152334	3113334	5610
GSE43294	GSM1060208	TAF15	Temporal cortices	None	HITS-CLIP	96941	38549	166
GSE43308	GSM1060382	FUS	Temporal cortices	None	HITS-CLIP	69393	54441	168
GSE43308	GSM1060383	FUS	Temporal cortices	None	HITS-CLIP	13968	6931	35
GSE43666	GSM1067861	NOP58	HEK293	None	PAR-CLIP	3885409	2861229	3371
GSE43666	GSM1067862	NOP58	HEK293	None	PAR-CLIP	3826346	2726870	2361
GSE43666	GSM1067863	NOP56	HEK293	None	PAR-CLIP	3921340	2522117	6380
GSE43666	GSM1067864	FBL	HEK293	None	PAR-CLIP	4535346	3134767	3467
GSE43666	GSM1067865	FBL	HEK293	None	PAR-CLIP	13102198	8146883	3725
GSE43801	GSM1071441	ORF1	HEK293T	None	PAR-CLIP	86119122	27325328	29854
GSE43801	GSM1071442	ORF1	HEK293T	None	PAR-CLIP	83883258	26585543	29139
GSE44615	GSM1087848	LIN28A	HEK293	None	PAR-CLIP	22355341	8679890	4985
GSE44615	GSM1087849	LIN28B	HEK293	None	PAR-CLIP	297954	154208	770
GSE44615	GSM1087850	LIN28B	HEK293	None	PAR-CLIP	1466194	589089	3366
GSE44615	GSM1087851	LIN28B	HEK293	None	PAR-CLIP	25171830	7736088	13154
GSE46705	GSM1135015	METTL14	HeLa	M14 overexpression	PAR-CLIP	25934	10581	5
GSE47502	GSM1151219	TOP3B	HeLa	None	HITS-CLIP	7887160	7160447	3257
GSE48245	GSM1173263	MOV10	HeLa	None	PAR-CLIP	791612	395048	952
GSE48245	GSM1173264	MOV10	HEK293	None	PAR-CLIP	549526	281758	713
GSE48245	GSM1173265	MOV10	HEK293	None	PAR-CLIP	263207	129808	279
GSE48245	GSM1173266	UPF1	HEK293	None	PAR-CLIP	3144575	1235813	3138
GSE49339	GSM1197605	YTHDF2	HeLa	None	PAR-CLIP	16408254	9886041	706
GSE49339	GSM1197607	YTHDF2	HeLa	None	PAR-CLIP	156365	90628	87
GSE50989	GSM1234283	ELAVL1	HEK293	None	PAR-CLIP	33915981	11378259	6772
GSE50989	GSM1234284	ELAVL1	HEK293	None	PAR-CLIP	76318821	32585602	175478
GSE53184	GSM1286117	ZFP36	HEK293/HEK293T	None	PAR-CLIP	2223832	783786	1602
GSE53554	GSM1296147	CTCF	U2OS	None	PAR-CLIP	10132989	8917825	19195
GSE53554	GSM1296148	CTCF	U2OS	None	PAR-CLIP	9670251	8632245	21379
GSE53554	GSM1296149	CTCF	U2OS	None	PAR-CLIP	19803240	17550070	39558
GSE19323	GSM480476	PTBP1	Hela	None	HITS-CLIP	1987442	1109744	4065
GSE19323	GSM480477	PTBP1	Hela	None	HITS-CLIP	2050963	1163995	4450
GSE19323	GSM480478	PTBP1	Hela	None	HITS-CLIP	1605056	941811	3450
GSE21918	GSM545206	IGF2BP1	HEK293	None	PAR-CLIP	8134623	4845085	19255
GSE21918	GSM545207	IGF2BP1	HEK293	None	PAR-CLIP	3155915	1994205	5904
GSE21918	GSM545208	IGF2BP2	HEK293	None	PAR-CLIP	9068999	5449885	24814
GSE21918	GSM545209	IGF2BP3	HEK293	None	PAR-CLIP	11320060	7088817	28515
GSE21918	GSM545210	PUM2	HEK293	None	PAR-CLIP	2454742	1268913	3960
GSE21918	GSM545211	QKI	HEK293	None	PAR-CLIP	4980975	2074728	3934
GSE21918	GSM545218	TNRC6A	HEK293	None	PAR-CLIP	1683676	415847	402
GSE21918	GSM545219	TNRC6B	HEK293	None	PAR-CLIP	1201206	402139	353
GSE21918	GSM545220	TNRC6C	HEK293	None	PAR-CLIP	1391590	620965	407
GSE23694	GSM581051	HNRNPH1	HEK293T	None	HITS-CLIP	2880388	1899254	26373
GSE23694	GSM581052	HNRNPH1	HEK293T	None	HITS-CLIP	9000010	5149635	59549
GSE28859	GSM714635	ELAVL1	HEK293	None	HITS-CLIP	9641918	3992524	30928
GSE28859	GSM714636	ELAVL1	HEK293	None	HITS-CLIP	9751768	3993502	29489
GSE28859	GSM714637	ELAVL1	HEK293	None	PAR-CLIP	5860813	3249914	13917
GSE28859	GSM714638	ELAVL1	HEK293	None	PAR-CLIP	4812370	2591383	10933
GSE28859	GSM714639	ELAVL1	HEK293	None	PAR-CLIP	18460488	12382033	76206
GSE28859	GSM714640	ELAVL1	HEK293	None	PAR-CLIP	18621978	10611202	47903
GSE28859	GSM714641	ELAVL1	HEK293	None	PAR-CLIP	10365541	7770232	27809
GSE29779	GSM738185	ELAVL1	HEK293	None	PAR-CLIP	8189999	4029154	21160
GSE29943	GSM741173	ELAVL1	HeLa	culture medium DMEM	PAR-CLIP	6595786	1924317	3270
GSE29943	GSM741174	ELAVL1	HeLa	culture medium SILAC	PAR-CLIP	8494242	2333201	4624
GSE29943	GSM741175	ELAVL1	HeLa	culture medium SILAC	PAR-CLIP	8881376	3034197	1204
GSE34491	GSM850170	HNRNPU	HeLa	None	HITS-CLIP	9677326	7039170	57724
GSE34491	GSM850171	HNRNPU	HeLa	None	HITS-CLIP	58871368	42058169	118256
GSE34993	GSM859978	HNRNPA1	HEK293T	None	HITS-CLIP	926596	573111	1129
GSE34993	GSM859979	HNRNPA1	HEK293T	None	HITS-CLIP	1533165	1025444	1912
GSE34993	GSM859980	HNRNPA1	HEK293T	None	HITS-CLIP	1470282	988709	3018
GSE34993	GSM859982	HNRNPA2B1	HEK293T	None	HITS-CLIP	17965720	15721934	8145
GSE34993	GSM859984	HNRNPF	HEK293T	None	HITS-CLIP	2874613	2207965	7361
GSE34993	GSM859985	HNRNPF	HEK293T	None	HITS-CLIP	4886336	3724549	7453
GSE34993	GSM859986	HNRNPF	HEK293T	None	HITS-CLIP	16667957	14203448	4902
GSE34993	GSM859987	HNRNPM	HEK293T	None	HITS-CLIP	3822498	2592080	2589
GSE34993	GSM859988	HNRNPM	HEK293T	None	HITS-CLIP	3127860	2277843	3963
GSE34993	GSM859990	HNRNPU	HEK293T	None	HITS-CLIP	1854289	1445671	4914
GSE34993	GSM859991	HNRNPU	HEK293T	None	HITS-CLIP	1242958	949149	2312
GSE34993	GSM859992	HNRNPU	HEK293T	None	HITS-CLIP	7353191	5577041	6735
GSE35799	GSM875073	HNRNPA2B1	MDA-MB-231	None	HITS-CLIP	3218421	1821624	14689
GSE35799	GSM875074	HNRNPA2B1	MDA-MB-231	None	HITS-CLIP	4294395	2410311	20524
GSE36987	GSM908088	PAPD5	HEK293	None	PAR-CLIP	4145662	1474008	2058
GSE36987	GSM908089	PAPD5	HEK293	None	PAR-CLIP	2579713	1043929	426
GSE37398	GSM917661	NUDT21	HEK293	None	PAR-CLIP	9744497	6074665	18826
GSE37398	GSM917664	CPSF6	HEK293	None	PAR-CLIP	9825099	5647015	33395
GSE37398	GSM917665	CPSF6	HEK293	None	PAR-CLIP	12978107	8229412	45497
GSE37398	GSM917666	CPSF4	HEK293	None	PAR-CLIP	6019092	2893738	3174
GSE37398	GSM917667	CPSF4	HEK293	None	PAR-CLIP	4716944	1965870	10775
GSE37398	GSM917668	CPSF3	HEK293	None	PAR-CLIP	17277849	8994406	21400
GSE37398	GSM917669	CPSF3	HEK293	None	PAR-CLIP	7044025	2217316	12290
GSE37398	GSM917670	CPSF2	HEK293	None	PAR-CLIP	4439441	1433573	2638
GSE37398	GSM917672	CPSF1	HEK293	None	PAR-CLIP	3708426	2116663	17537
GSE37398	GSM917674	FIP1L1	HEK293	None	PAR-CLIP	9180285	6952093	25334
GSE37398	GSM917675	FIP1L1	HEK293	None	PAR-CLIP	11419271	6569685	22898
GSE37398	GSM917676	CSTF2	HEK293	None	PAR-CLIP	9616959	7637531	68009
GSE37398	GSM917677	CSTF2T	HEK293	None	PAR-CLIP	21864404	18205915	141444
GSE37398	GSM917678	CSTF2T	HEK293	None	PAR-CLIP	11237805	3061782	9469
GSE37524	GSM921128	MOV10	HEK293	culture medium DMEM	PAR-CLIP	18055005	8886247	55567
GSE38157	GSM936506	ALKBH5	HEK293	culture medium DMEM	PAR-CLIP	866992	429228	3695
GSE38157	GSM936507	C17orf85	HEK293	culture medium DMEM	PAR-CLIP	1932134	904153	1213
GSE38157	GSM936508	RTCB	HEK293	culture medium DMEM	PAR-CLIP	2460660	1311386	10917
GSE38157	GSM936509	CAPRIN1	HEK293	culture medium DMEM	PAR-CLIP	6106154	3965172	27034
GSE38157	GSM936510	ZC3H7B	HEK293	culture medium DMEM	PAR-CLIP	8253950	5992046	48173
GSE39086	GSM955511	DGCR8	HEK293T	None	HITS-CLIP	187988	87914	657
GSE39086	GSM955512	DGCR8	HEK293T	None	HITS-CLIP	2094767	708946	1889
GSE39086	GSM955513	DGCR8	HEK293T	None	HITS-CLIP	431171	184637	947
GSE39682	GSM977615	FMR1	HEK293/HEK295T	None	PAR-CLIP	10464938	6045652	18963
GSE39682	GSM977616	FMR1	HEK293/HEK294T	None	PAR-CLIP	7561219	4065314	21649
GSE39682	GSM977617	FMR1	HEK293/HEK293T	None	PAR-CLIP	106401092	65696423	218507
GSE39682	GSM977618	FMR1	HEK293	None	PAR-CLIP	127508527	81456621	264969
GSE39682	GSM977620	FXR2	HEK293/HEK296T	None	PAR-CLIP	2970898	1184059	8417
GSE39872	GSM980593	LIN28A	H9	None	HITS-CLIP	7545901	4412323	25923
GSE39872	GSM980594	LIN28A	HEK293	None	HITS-CLIP	5652060	2303769	17329
GSE40651	GSM998875	FUS	tissue: brain	None	HITS-CLIP	73357694	59709533	152128
SRA025082	SRR070448	FUS	HEK293	None	PAR-CLIP	2727240	1656381	9483
SRA025082	SRR070460	EWSR1	HEK293	None	PAR-CLIP	2429552	1486311	12714
SRA025082	SRR070462	TAF15	HEK293	None	PAR-CLIP	1495382	1026679	4949
